# Supplementary figures and images for: Insights from the transcriptome and metabolome into the molecular basis of diapause in Leguminivora glycinivorella (Lepidoptera, Olethreutidae)
Source: PLoS One. 2025 Jun 4;20(6):e0322332. doi: 10.1371/journal.pone.0322332 (PMC12136294; doi:10.1371/journal.pone.0322332)

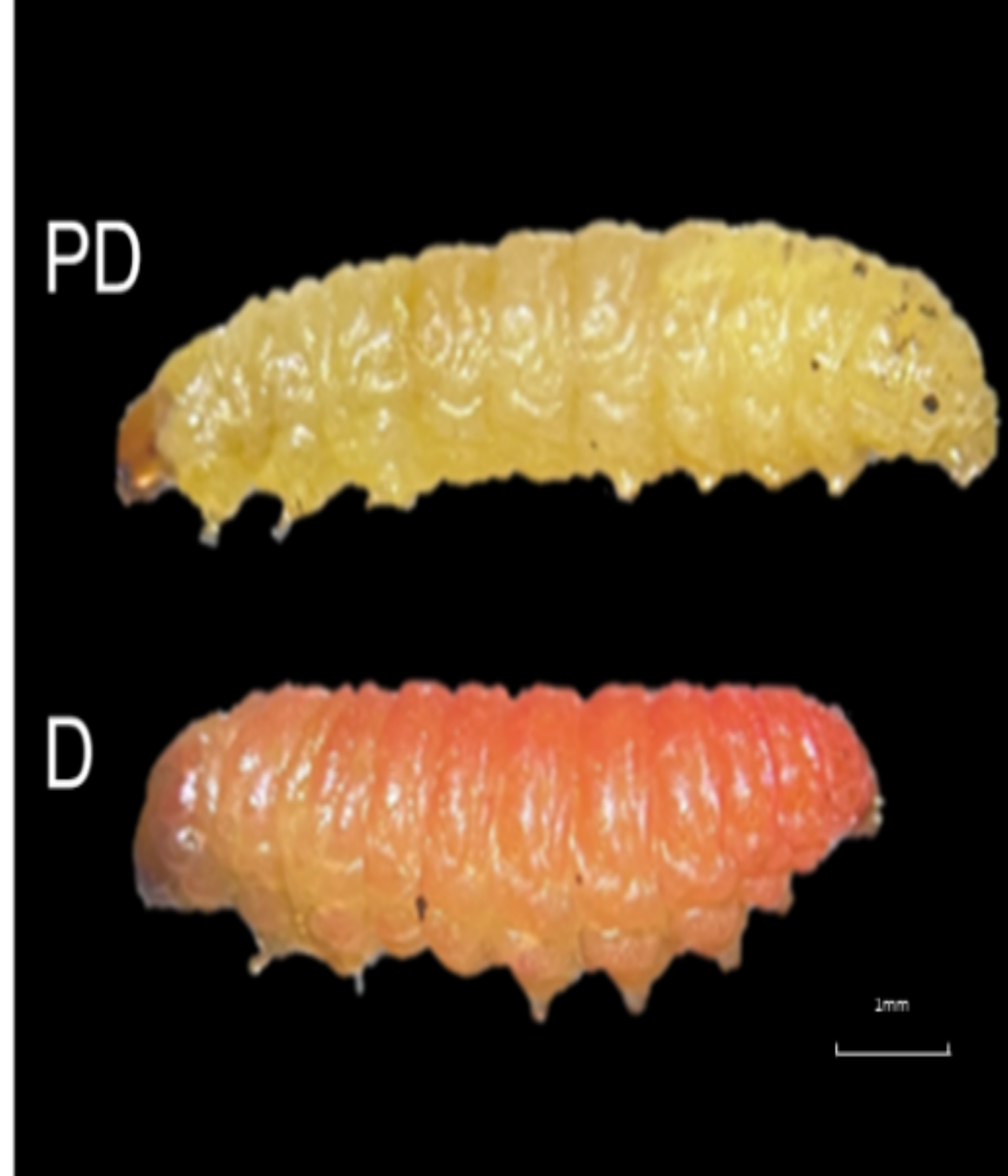

Supplement: S1 Fig — (TIF) [file pone.0322332.s001.tif]

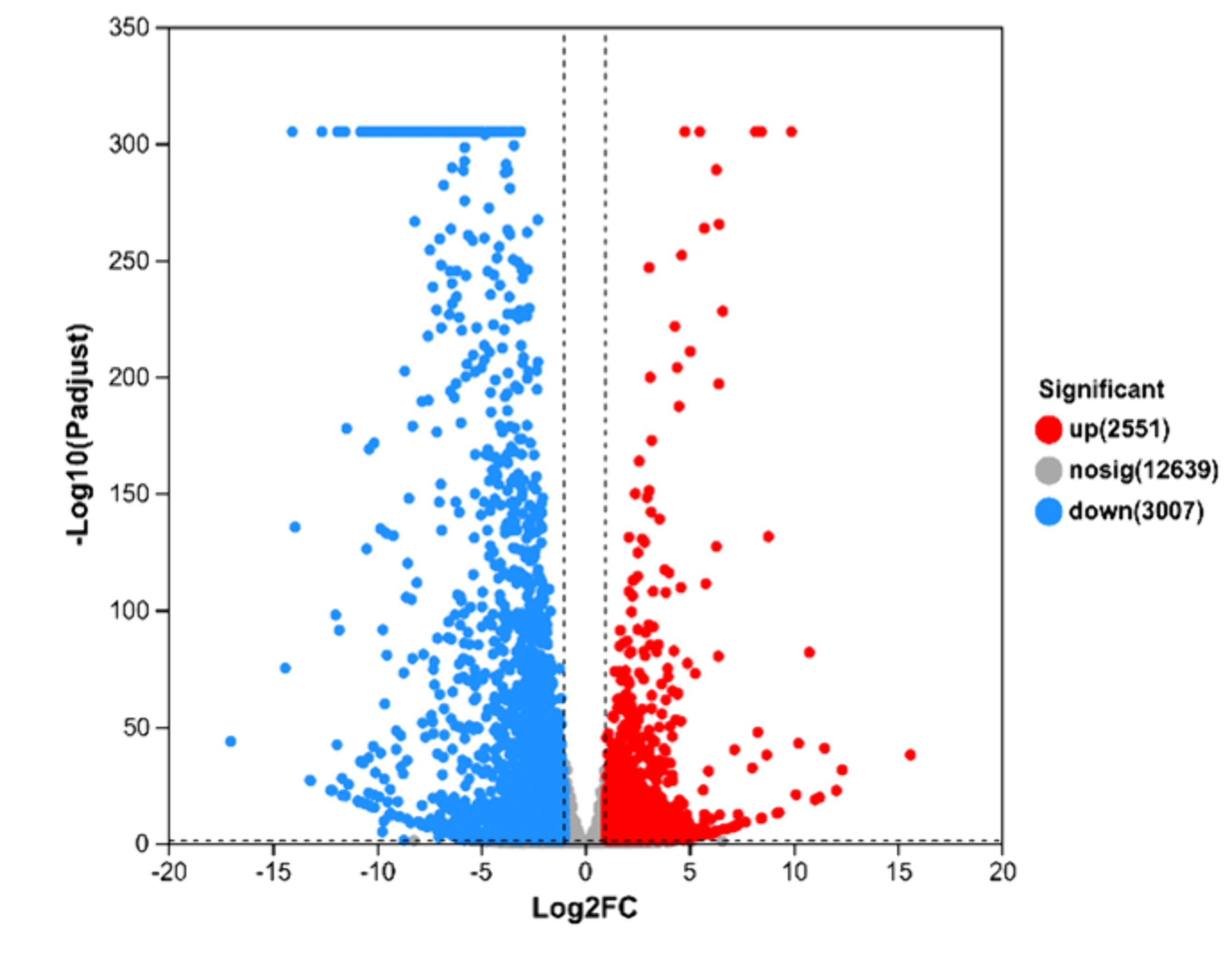

Supplement: S2 Fig — (TIF) [file pone.0322332.s002.tif]

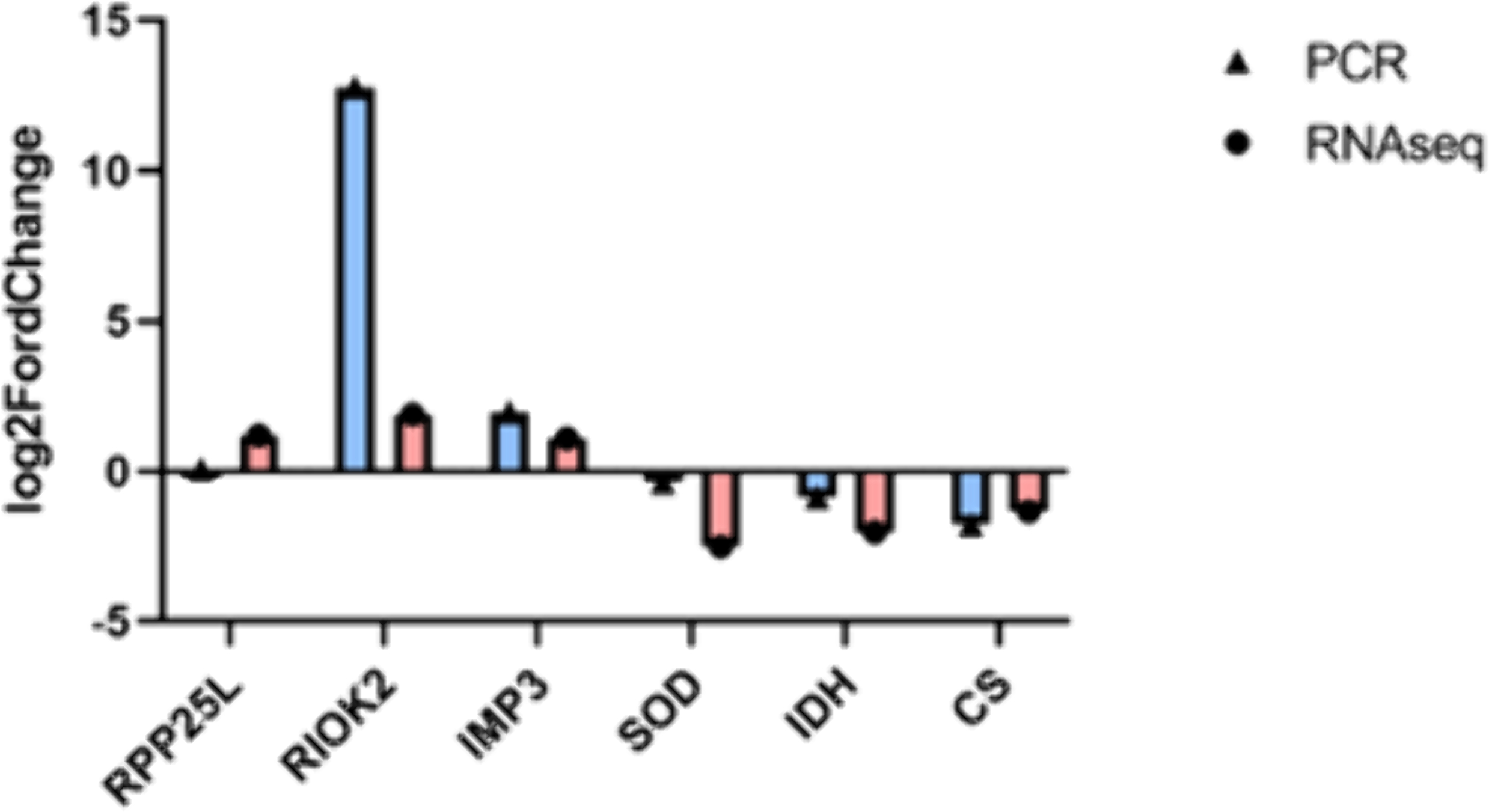

Supplement: S3 Fig — (TIF) [file pone.0322332.s003.tif]
