# Supplementary material for: Insights from the transcriptome and metabolome into the molecular basis of diapause in Leguminivora glycinivorella (Lepidoptera, Olethreutidae)
Source: PLoS One. 2025 Jun 4;20(6):e0322332. doi: 10.1371/journal.pone.0322332 (PMC12136294; doi:10.1371/journal.pone.0322332)
Supplement: S1 Table — (DOCX) [file pone.0322332.s004.docx]

**Supporting Information--Tables**

**Supporting Information** **S1 Table.** Identification information of metabolites of *L.glycinivorella.*

| Serial number | Metabolite | M/Z | Intensity | Retention time | CV% | ppm Error | Fold Change | p-value |
| --- | --- | --- | --- | --- | --- | --- | --- | --- |
| Amino acids and their derivatives | | | | | | | | |
| 1 | 2-Aminobenzoic Acid | 120.04 | 4.4 | 2.43 | 11.58 | 0.85 | 1.24 | ** |
| 2 | 2-Keto-6-acetamidocaproate | 229.12 | 3.67 | 3.19 | 22.68 | 1.77 | 0.64 | ** |
| 3 | 3-Hydroxy-N6,N6,N6-trimethyl-L-lysine | 205.15 | 4.25 | 0.57 | 8.32 | 3.55 | 0.86 | ** |
| 4 | 5-Hydroxy-L-tryptophan | 262.12 | 3.26 | 2.74 | 28.32 | 0.64 | 1.68 | ** |
| 5 | L-Aspartate-semialdehyde | 159.08 | 5.21 | 0.62 | 13.34 | 1.34 | 1.28 | ** |
| 6 | N-Acetyl-1-aspartylglutamic acid | 303.08 | 3.63 | 1.99 | 26.44 | 0.46 | 1.63 | ** |
| 7 | N-Acetyl-L-aspartic acid | 176.06 | 3.95 | 1.07 | 20.47 | 0.95 | 0.67 | ** |
| 8 | N6-Acetyl-L-lysine | 187.11 | 4.48 | 3.45 | 11.4 | 3.15 | 0.8 | ** |
| 9 | Saccharopine | 277.14 | 4.52 | 0.62 | 10.18 | 1.36 | 0.82 | ** |
| Nucleotides and their derivatives | | | | | | | | |
| 10 | Adenosine | 268.1 | 4.59 | 0.7 | 23.74 | 1.69 | 1.55 | ** |
| 11 | Adenylosuccinate | 462.07 | 6.96 | 2.71 | 6.08 | 0.53 | 0.89 | ** |
| 12 | Adenylsuccinic acid | 462.07 | 4.71 | 0.71 | 17.31 | 1.06 | 0.72 | * |
| 13 | CDP-ethanolamine | 445.05 | 4.89 | 0.63 | 8.73 | 1.4 | 0.85 | ** |
| Hormones and signaling molecules | | | | | | | | |
| 14 | Endomorphin-2 | 594.27 | 4.85 | 3.86 | 14.05 | 0.02 | 1.3 | ** |
| 15 | Norepinephrine | 214.07 | 3.99 | 3.69 | 24.86 | 2.31 | 1.58 | ** |
| 16 | Normetanephrine | 164.07 | 4.05 | 1.01 | 11.93 | 3.95 | 1.25 | ** |
| 17 | P-Octopamine | 152.07 | 3.79 | 1.35 | 20 | 5.18 | 1.45 | ** |
| Lipids (glycerolipids) | | | | | | | | |
| 18 | DG(15:0/18:3(6Z,9Z,12Z)/0:0) | 599.46 | 5.15 | 7.34 | 46.55 | 1.73 | 0.37 | * |
| 19 | DG(15:0/18:4(6Z,9Z,12Z,15Z)/0:0) | 597.45 | 4.65 | 7.18 | 43.41 | 1.55 | 0.4 | ** |
| 20 | LysoPC(20:0/0:0) | 596.37 | 4.7 | 6.18 | 27.03 | 4.04 | 0.58 | ** |
| 21 | LysoPC(22:4(7Z,10Z,13Z,16Z)/0:0) | 536.35 | 6.76 | 6.3 | 17.04 | 4.35 | 0.72 | ** |
| 22 | PA(8:0/a-13:0) | 517.29 | 3.78 | 6.23 | 59.14 | 0.28 | 0.25 | ** |
| 23 | PA(8:0/a-15:0) | 586.35 | 3.87 | 5.89 | 40.96 | 0.46 | 0.42 | ** |
| 24 | PC(22:4(7Z,10Z,13Z,16Z)/18:2(9Z,12Z)) | 816.59 | 5.13 | 6.69 | 14.8 | 3.63 | 0.76 | * |
| 25 | PE(15:0/22:4(7Z,10Z,13Z,16Z)) | 736.53 | 4.96 | 7.62 | 17.6 | 6.49 | 1.38 | ** |
| 26 | PE-NMe2(15:0/20:3(8Z,11Z,14Z)) | 800.54 | 5.93 | 6.5 | 10.5 | 0.57 | 1.21 | ** |
| 27 | PG(18:0/18:2(9Z,12Z)) | 816.57 | 4.86 | 6.76 | 18.34 | 1.61 | 1.4 | ** |
| 28 | PS(15:0/24:1(15Z)) | 830.59 | 4.5 | 6.51 | 17.08 | 0.7 | 1.31 | * |
| Lipids (sphingolipids) | | | | | | | | |
| 29 | SM(d18:1/12:0) | 669.49 | 4.55 | 7.62 | 21.61 | 1.55 | 1.48 | ** |
| 30 | Sphingosine | 300.29 | 4.83 | 6.09 | 25.59 | 1.49 | 1.6 | ** |
| Lipids (fatty acid derivatives) | | | | | | | | |
| 31 | 11-beta-Hydroxyandrosterone-3-glucuronide | 482.25 | 3.14 | 6.16 | 74.27 | 2.9 | 4.19 | * |
| 32 | 13(S)-HpODE | 311.22 | 4.69 | 6.85 | 23.68 | 0.19 | 0.63 | ** |
| 33 | 16-Hydroxyhexadecanoic acid | 290.27 | 5.21 | 6.06 | 9.68 | 1.82 | 0.83 | ** |
| 34 | 18-hydroxyoleate | 593.48 | 4.8 | 6.71 | 20.62 | 1.26 | 0.67 | ** |
| 35 | Alpha-Dimorphecolic acid | 279.23 | 4.47 | 7.51 | 15.22 | 1.27 | 0.75 | ** |
| 36 | Vanillylmandelic acid | 181.05 | 4.25 | 3.15 | 40.56 | 0.8 | 0.43 | ** |
| Organic acid | | | | | | | | |
| 37 | 2-Hydroxy-3-(4-hydroxyphenyl)propenoic acid | 222.08 | 4.59 | 2.17 | 12.89 | 1.17 | 0.78 | ** |
| 38 | 2-Propylglutaric acid | 216.12 | 3.48 | 3.3 | 36.09 | 1.49 | 1.95 | ** |
| 39 | 3,4-Dihydroxyphenylacetaldehyde | 363.11 | 4.06 | 4.69 | 21.5 | 0.15 | 0.66 | ** |
| 40 | 3-Hydroxyoctanoic acid | 479.32 | 3.57 | 6.03 | 45.06 | 1.27 | 2.32 | ** |
| 41 | 3-Isopropylmalic acid | 218.1 | 3.73 | 2 | 16.92 | 0.78 | 0.72 | ** |
| 42 | 4-Hydroxyphenylpyruvic acid | 179.03 | 3.75 | 5.9 | 45.18 | 3.47 | 2.33 | ** |
| 43 | Citric acid | 215.02 | 5.81 | 1.22 | 6.83 | 1.15 | 0.88 | ** |
| 44 | Glutaric acid | 174.08 | 5.06 | 0.7 | 9.32 | 0.92 | 0.84 | ** |
| 45 | Homovanillic acid | 224.09 | 3.48 | 3.59 | 36.17 | 1.37 | 1.95 | ** |
| 46 | Ketoleucine | 129.05 | 5.76 | 4.21 | 7.23 | 7.26 | 0.87 | ** |
| 47 | Pyruvic Acid | 175.02 | 3.91 | 0.72 | 18.96 | 3.95 | 0.69 | ** |
| 48 | Quinoline-4,8-diol | 162.05 | 5.09 | 3.16 | 8.17 | 0.89 | 1.16 | ** |
| Indole metabolites | | | | | | | | |
| 49 | 5-Hydroxyindoleacetate | 192.07 | 3.5 | 3.49 | 31.03 | 0.89 | 1.77 | ** |
| 50 | 5-Hydroxyindoleacetic acid | 209.09 | 6.26 | 2.43 | 7.91 | 1.06 | 1.16 | ** |
| 51 | 5-Hydroxyindoleacetylglycine | 249.09 | 5.05 | 4.17 | 29.23 | 1.46 | 1.71 | ** |
| 52 | Indole-3-acetic Acid | 158.06 | 4.35 | 2.05 | 17.41 | 0.47 | 1.38 | ** |
| 53 | Kynurenic Acid | 188.03 | 4.28 | 4.18 | 11.01 | 2.97 | 1.23 | ** |
| 54 | Xanthurenic Acid | 206.04 | 4.24 | 3.11 | 19.5 | 1.05 | 1.43 | ** |
| Vitamins and their derivatives | | | | | | | | |
| 55 | Biotin | 243.08 | 5.11 | 4.63 | 7.95 | 0.47 | 1.16 | ** |
| 56 | L-Ascorbic acid | 177.04 | 4.54 | 1.04 | 14.78 | 0.78 | 1.31 | ** |
| 57 | Pantothenic Acid | 218.1 | 6.41 | 3.43 | 7.33 | 1.54 | 1.15 | ** |
| 58 | Pyridoxal | 168.07 | 4.36 | 2.87 | 13.7 | 0.51 | 1.29 | ** |
| other | | | | | | | | |
| 59 | Atropaldehyde | 133.06 | 4.04 | 5.8 | 19.29 | 0.37 | 1.43 | ** |
| 60 | Morph | 292.13 | 5.33 | 3.55 | 14.08 | 8.56 | 1.3 | ** |

Note: P<0.05 is represented by *, P<0.01 is represented by **.
