# Supplementary material for: Insights from the transcriptome and metabolome into the molecular basis of diapause in Leguminivora glycinivorella (Lepidoptera, Olethreutidae)
Source: PLoS One. 2025 Jun 4;20(6):e0322332. doi: 10.1371/journal.pone.0322332 (PMC12136294; doi:10.1371/journal.pone.0322332)
Supplement: S2 Table — (DOCX) [file pone.0322332.s005.docx]

**Supporting Information S2 Table.** List of primers used for quantitative real-time PCR

| Unigene ID | Forward primer |
| --- | --- |
| actin-F | 5' TCTTCCAGCCCTCGTTCC 3' |
| actin-R | 5' TTGTTGGCATACAGGTCCTTAC 3' |
| SOD-F | 5' CCAGACCTGCCGTATGAG 3' |
| SOD-R | 5' TGTCAATGTCGCCTTTAGC 3' |
| CS-F | 5' AGTTCGCCCTGAAGCACC 3' |
| CS-R | 5' CAGCCCGTAATACTGTAGAAGG 3' |
| IDH-F | 5' GGAAAGGTGGAGTTGGTGTA 3' |
| IDH-R | 5' CCGTCATATCGCTTGAGAAT 3' |
| IMP3-F | 5' CCGTGAGGTAGCGAACAA 3' |
| IMP3-R | 5' TGACCGGCTGACCAGGAA 3' |
| RIOK2-F | 5' CGTCCATTGCGAATCTCC 3' |
| RIOK2-R | 5' CCACGCCTATCTGGTTGC 3' |
| RPP26L-F | 5' TTGTAAGCGAGTTTGGTA 3' |
| RPP25L-R | 5' AAAGGTGGAAGTAAGATG 3' |
