# Supplementary material for: Insights from the transcriptome and metabolome into the molecular basis of diapause in Leguminivora glycinivorella (Lepidoptera, Olethreutidae)
Source: PLoS One. 2025 Jun 4;20(6):e0322332. doi: 10.1371/journal.pone.0322332 (PMC12136294; doi:10.1371/journal.pone.0322332)
Supplement: S3 Table — (DOCX) [file pone.0322332.s006.docx]

**Supporting Information S3 Table.** Sequencing data results of diapause and pre-diapause of *L.glycinivorella*.

| Sample | Raw reads | Raw bases | Clean reads | Clean bases | Error rate(%) | Q20(%) | Q30(%) | GC content(%) |
| --- | --- | --- | --- | --- | --- | --- | --- | --- |
| PD_1 | 49234768 | 7.434E+09 | 48735708 | 7238793744 | 0.0125 | 98.45 | 95.24 | 50.83 |
| PD_2 | 47201108 | 7.127E+09 | 46695292 | 6954573752 | 0.0124 | 98.45 | 95.28 | 52.6 |
| PD_3 | 49451336 | 7.467E+09 | 48803704 | 7187381312 | 0.0125 | 98.43 | 95.2 | 52.72 |
| D_1 | 42934998 | 6.483E+09 | 42558612 | 6360937925 | 0.0125 | 98.41 | 95.08 | 47.37 |
| D_2 | 53848698 | 8.131E+09 | 53029038 | 7910677513 | 0.0123 | 98.53 | 95.48 | 48.12 |
| D_3 | 43636080 | 6.589E+09 | 43144608 | 6450326144 | 0.0128 | 98.28 | 94.69 | 46.75 |
