# Supplementary material for: Insights from the transcriptome and metabolome into the molecular basis of diapause in Leguminivora glycinivorella (Lepidoptera, Olethreutidae)
Source: PLoS One. 2025 Jun 4;20(6):e0322332. doi: 10.1371/journal.pone.0322332 (PMC12136294; doi:10.1371/journal.pone.0322332)
Supplement: S4 Table — (DOCX) [file pone.0322332.s007.docx]

**Supporting Information S4 Table.** Summary of the unigenes annotated in different databases for diapause and pre-diapause of *L.glycinivorella*.

|  | Expre_Gene number（percent） | Expre_Transcript number（percent） | All_Gene number（percent） | All_Transcript number（percent） |
| --- | --- | --- | --- | --- |
| GO | 7553(0.4606) | 10059(0.4293) | 7939(0.4363) | 11001(0.4104) |
| KEGG | 8140(0.4964) | 12543(0.5353) | 8331(0.4578) | 13586(0.5068) |
| EggNOG | 14172(0.8643) | 20681(0.8826) | 14670(0.8062) | 22514(0.8399) |
| NR | 15662(0.9551) | 22641(0.9663) | 16726(0.9192) | 25244(0.9417) |
| Swiss-Prot | 10298(0.628) | 15378(0.6563) | 10507(0.5774) | 16581(0.6186) |
| Pfam | 12081(0.7367) | 17709(0.7558) | 12366(0.6796) | 19086(0.712) |
| Total_anno | 15666(0.9554) | 22645(0.9665) | 16731(0.9194) | 25250(0.942) |
| Total | 16398(1.0) | 23431(1.0) | 18197(1) | 26806(1) |
