# Supplementary material for: Insights from the transcriptome and metabolome into the molecular basis of diapause in Leguminivora glycinivorella (Lepidoptera, Olethreutidae)
Source: PLoS One. 2025 Jun 4;20(6):e0322332. doi: 10.1371/journal.pone.0322332 (PMC12136294; doi:10.1371/journal.pone.0322332)
Supplement: S5 Table — (DOCX) [file pone.0322332.s008.docx]

**Supporting Information S5 Table.** Differentially expressed genes in the top 10 differences between diapausing phase and pre-diapausing phase of *L.glycinivorella.*

|  | Gene_id | Gene description | Log2FC | Pvalue |
| --- | --- | --- | --- | --- |
| up-regulated genes | gene-LOC125226433 | lipase 1-like | 6.10904154 | 4.21E-06 |
|  | gene-LOC125228902 | cAMP-dependent protein kinase catalytic subunit gamma-like | 5.43218957 | 0.000116093 |
|  | gene-LOC125228903 | cAMP-dependent protein kinase catalytic subunit alpha-like | 3.448800481 | 0.009720711 |
|  | gene-LOC125241857 | enolase-like, transcript variant X2 | 3.070643737 | 5.82E-09 |
|  | gene-LOC125228904 | cAMP-dependent protein kinase catalytic subunit alpha-like | 2.956586358 | 0.02558121 |
|  | gene-LOC125236911 | venom peptide isomerase heavy chain-like | 2.924081795 | 0.012441721 |
|  | gene-LOC125232182 | palmitoyl-protein thioesterase 1-like | 2.904839239 | 1.49E-24 |
|  | gene-LOC125232017 | mismatch repair endonuclease PMS2 | 2.797453763 | 5.25E-15 |
|  | gene-LOC125226968 | calcium/calmodulin-dependent 3',5'-cyclic nucleotide phosphodiesterase 1 | 2.772072485 | 5.97E-20 |
|  | gene-LOC125231148 | serine/threonine-protein kinase RIO3-like | 2.707486088 | 2.82E-12 |
| down-regulated genes | gene-LOC125235932 | acidic mammalian chitinase-like, transcript variant X1 | -11.4880399 | 4.17E-22 |
|  | gene-LOC125236114 | acidic mammalian chitinase-like | -10.1235831 | 4.59E-174 |
|  | gene-LOC125235902 | acidic mammalian chitinase-like, transcript variant X1 | -9.61014973 | 6.71E-62 |
|  | gene-LOC125235905 | acidic mammalian chitinase-like, transcript variant X1 | -9.35097073 | 6.27E-25 |
|  | gene-LOC125235918 | acidic mammalian chitinase-like, transcript variant X1 | -8.72956919 | 1.23E-31 |
|  | gene-LOC125230532 | alpha-amylase 2-like | -8.37627651 | 0 |
|  | gene-LOC125225741 | acyl-CoA Delta(11) desaturase-like | -7.85953721 | 0 |
|  | gene-LOC125229584 | - | -7.59364409 | 6.72E-09 |
|  | gene-LOC125233774 | probable alpha-ketoglutarate-dependent hypophosphite dioxygenase | -7.17807431 | 0 |
|  | gene-LOC125235952 | acidic mammalian chitinase-like | -7.11036669 | 3.73E-07 |
