# Supplementary material for: Insights from the transcriptome and metabolome into the molecular basis of diapause in Leguminivora glycinivorella (Lepidoptera, Olethreutidae)
Source: PLoS One. 2025 Jun 4;20(6):e0322332. doi: 10.1371/journal.pone.0322332 (PMC12136294; doi:10.1371/journal.pone.0322332)
Supplement: S6 Table — (DOCX) [file pone.0322332.s009.docx]

**Supporting Information S6 Table.** The most enriched GO terms of the DEGs between the diapause and pre-diapause of *L.glycinivorella*.

|  | DEGene Number | GO accession number | GO Term | Description |
| --- | --- | --- | --- | --- |
| Up-DEG | 52 | GO:1901363 | MF | heterocyclic compound binding |
|  | 52 | GO:0097159 | MF | organic cyclic compound binding |
|  | 15 | GO:0003723 | MF | RNA binding |
|  | 37 | GO:0003676 | MF | nucleic acid binding |
|  | 5 | GO:0003899 | MF | DNA-directed 5'-3' RNA polymerase activity |
|  | 11 | GO:0030880 | CC | RNA polymerase complex |
|  | 11 | GO:0000428 | CC | DNA-directed RNA polymerase complex |
|  | 13 | GO:0005730 | CC | nucleolus |
|  | 35 | GO:0032991 | CC | protein-containing complex |
|  | 15 | GO:1990904 | CC | ribonucleoprotein complex |
|  | 31 | GO:0016070 | BP | RNA metabolic process |
|  | 42 | GO:0090304 | BP | nucleic acid metabolic process |
|  | 43 | GO:0006139 | BP | nucleobase-containing compound metabolic process |
|  | 43 | GO:0046483 | BP | heterocycle metabolic process |
|  | 43 | GO:0006725 | BP | cellular aromatic compound metabolic process |
| DownDEG- | 138 | GO:0003824 | MF | catalytic activity |
|  | 26 | GO:0016798 | MF | hydrolase activity, acting on glycosyl bonds |
|  | 48 | GO:0016491 | MF | oxidoreductase activity |
|  | 22 | GO:0004553 | MF | hydrolase activity, hydrolyzing O-glycosyl compounds |
|  | 16 | GO:0008061 | MF | chitin binding |
|  | 7 | GO:0005777 | CC | peroxisome |
|  | 7 | GO:0042579 | CC | microbody |
|  | 1 | GO:0036125 | CC | fatty acid beta-oxidation multienzyme complex |
|  | 1 | GO:0005782 | CC | peroxisomal matrix |
|  | 1 | GO:0045254 | CC | pyruvate dehydrogenase complex |
|  | 42 | GO:0005975 | BP | carbohydrate metabolic process |
|  | 52 | GO:0044281 | BP | small molecule metabolic process |
|  | 42 | GO:0043436 | BP | oxoacid metabolic process |
|  | 42 | GO:0019752 | BP | carboxylic acid metabolic process |
|  | 42 | GO:0006082 | BP | organic acid metabolic process |
