# Supplementary material for: Insights from the transcriptome and metabolome into the molecular basis of diapause in Leguminivora glycinivorella (Lepidoptera, Olethreutidae)
Source: PLoS One. 2025 Jun 4;20(6):e0322332. doi: 10.1371/journal.pone.0322332 (PMC12136294; doi:10.1371/journal.pone.0322332)
Supplement: S7 Table — (DOCX) [file pone.0322332.s010.docx]

**Supporting Information S7 Table.** The top10 enriched KEGG pathway of the DEGs between he diapause and pre-diapause of *L.glycinivorella*.

|  | Num | Pathway id | Description | Pvalue |
| --- | --- | --- | --- | --- |
| Up-regulated | 44 | map03008 | Ribosome biogenesis in eukaryotes | 1.45E-61 |
|  | 16 | map03018 | RNA degradation | 2.72E-17 |
|  | 14 | map03420 | Nucleotide excision repair | 8.54E-17 |
|  | 14 | map03020 | RNA polymerase | 1.90E-16 |
|  | 9 | map04623 | Cytosolic DNA-sensing pathway | 3.84E-11 |
|  | 7 | map04742 | Taste transduction | 1.78E-08 |
|  | 7 | map03410 | Base excision repair | 2.48E-08 |
|  | 6 | map03430 | Mismatch repair | 1.06E-07 |
|  | 7 | map05032 | Morphine addiction | 1.32E-06 |
|  | 4 | map04610 | Complement and coagulation cascades | 2.43E-06 |
| Down-regulated | 34 | map00010 | Glycolysis / Gluconeogenesis | 4.13E-46 |
|  | 34 | map00520 | Amino sugar and nucleotide sugar metabolism | 8.87E-40 |
|  | 36 | map04146 | Peroxisome | 1.82E-33 |
|  | 23 | map00380 | Tryptophan metabolism | 1.54E-29 |
|  | 30 | map04212 | Longevity regulating pathway - worm | 4.49E-26 |
|  | 20 | map00500 | Starch and sucrose metabolism | 4.50E-25 |
|  | 19 | map00071 | Fatty acid degradation | 4.46E-22 |
|  | 17 | map00052 | Galactose metabolism | 7.87E-20 |
|  | 17 | map00020 | Citrate cycle (TCA cycle) | 2.66E-19 |
|  | 17 | map00981 | Insect hormone biosynthesis | 1.40E-18 |
