# Supplementary material for: Insights from the transcriptome and metabolome into the molecular basis of diapause in Leguminivora glycinivorella (Lepidoptera, Olethreutidae)
Source: PLoS One. 2025 Jun 4;20(6):e0322332. doi: 10.1371/journal.pone.0322332 (PMC12136294; doi:10.1371/journal.pone.0322332)
Supplement: S8 Table — (DOCX) [file pone.0322332.s011.docx]

**Supporting Information S8 Table.** KEGG metabolic pathway and differentially expressed genes in *L.glycinivorella* during diapause.

| KEGG pathway | ID | Gene number | Differentially expressed genes | | P_value |
| --- | --- | --- | --- | --- | --- |
|  |  |  | up | down |  |
| Ribosome biogenesis in eukaryotes | map03008 | 44 | RPP25, IMP3, RIOK2, NOG1, UTP15, POP5, NHP2, NAN1, MPP10, REX1 |  | ** |
| RNA degradation | map03018 | 16 | RRP40, DCP2, LSM7, MTR3, ENO, WDR61 |  | ** |
| cleotide excision repair | map03420 | 14 | POLE3, XPC, TFIIH1, CETN2, ERCC4 |  | ** |
| RNA polymerase | map03020 | 14 | RPC8, RPA12, POLR2M, RPB1 |  | ** |
| Cytosolic DNA-sensing pathway | map04623 | 9 | TREX1, RPABC2, TMEM173, RPAC1 |  | ** |
| Glycolysis / Gluconeogenesis | map00010 | 34 |  | HK, FBP, GPI, PFK, PK, PGK, ALDH, ALDO, GAPDH, PGAM, MINPP1, ENO, PCK, DLAT, LDH, DLD, TPI | ** |
| Amino sugar and nucleotide sugar metabolism | map00520 | 34 |  | E3.2.1.14, HK, GMPP, CHS1, GPI, pgm, CYB5R, GMDS, HEXA_B, GALT, galK | ** |
| Peroxisome | map04146 | 36 |  | ACSL, CAT, E1.3.3.6, MPV17L, PEX7, XDH, HAO, FAR, ALDL1, PHYH, SOD, IDH1, XDH, FAR | ** |
| Tryptophan metabolism | map00380 | 23 |  | CAT, GCDH, TDO2, HADH, ALDH, KMO, HADHA, ECHS1, CCBL | ** |
| Longevity regulating pathway - worm | map04212 | 30 |  | CAT, SCD, VHL, GST, SMEK, FAR, IRS1, SOD2, DESAT1, GCLC, AKT, HSP, HIF1A | ** |
| Starch and sucrose metabolism | map00500 | 20 |  | HK, GPI, pgm, TPS, malZ, INV, AGL, G6PC, AGL, GYS, GAA, PYG, AMY | ** |
| Fatty acid degradation | map00071 | 19 |  | ACSL, GCDH, E1.3.3.6, ALDH, HADH, HADHA, ECHS1, ALDH7A1 | ** |
| Galactose metabolism | map00052 | 17 |  | HK, GALM, pgm, malZ, INV, G6PC, galK, PFK | ** |
| Citrate cycle (TCA cycle) | map00020 | 17 |  | LSC2, PDHB, MDH2, DLST, PC, SDHD, CS, IDH1, LSC1, ACLY, ACO, OGDH | ** |
| Insect hormone biosynthesis | map00981 | 17 |  | FOHSDR, ALDH, CYP18A1, JHEH, ALDH, SHD, FPPP | ** |

Note：P<0.05 is represented by *, P<0.01 is represented by **.

Noet：hexokinase type 2, HK; putative chitinase 1, E3.2.1.14; long-chain-fatty-acid--CoA ligase 5,ACSL; succinate--CoA ligase [ADP-forming] subunit beta, LSC2; catalase-like, CAT; pyruvate dehydrogenase E1 component subunit beta, PDHB; acyl-CoA Delta-9 desaturase-like, SCD; acidic mammalian chitinase-like, E3.2.1.14; glutaryl-CoA dehydrogenase, GCDH; peroxisomal acyl-coenzyme A oxidase 3, ACOX3; fructose-1,6-bisphosphatase 1, FBP; mpv17-like protein, MPV17L; tryptophan 2,3-dioxygenase, TDO2; pyruvate kinase-like, PK; aldehyde oxidase 4-like, XDH; chitin synthase chs-2, CHS1; malate dehydrogenase, mitochondrial, MDH2; glutathione S-transferase theta-1-like, GST; serine/threonine-protein phosphatase 4 regulatory subunit 3, SMEK; aldehyde dehydrogenase X, ALDH; glucose-6-phosphate isomerase, GPI; fatty acyl-CoA reductase wat-like, FAR; galactose mutarotase-like, GALM; cytochrome P450 18a1, CYP18A1; probable 3-hydroxyacyl-CoA dehydrogenase B0272.3, HADH; phosphoglucomutase, pgm; ATP-binding cassette sub-family D member-like, ABCD2; NADH-cytochrome b5 reductase 2, transcript variant X1, CYB5R; juvenile hormone epoxide hydrolase-like, JHEH; aldehyde dehydrogenase 1A1-like, ALDH; pyruvate carboxylase, PC; alpha,alpha-trehalose-phosphate synthase [UDP-forming]-like, TPS; maltase A1-like, malZ; peroxisomal multifunctional enzyme type 2-like, HSD17B4; GDP-mannose 4,6 dehydratase, transcript variant X1, GMDS; probable alpha-ketoglutarate-dependent hypophosphite dioxygenase, PHYH; superoxide dismutase [Mn], SOD2; fructose-bisphosphate aldolase, ALDO; probable citrate synthase 2, mitochondrial, CS; peroxiredoxin-5, mitochondrial, PRDX5; acyl-CoA Delta(11) desaturase-like, DESAT1; phosphatidylinositol 4,5-bisphosphate 3-kinase catalytic subunit delta isoform, PIK3CA_B_D; triosephosphate isomerase, TPI; beta-hexosaminidase subunit alpha-like, HEXA_B; glutathione S-transferase 1-like, transcript variant X1, GST; tropinone reductase-like 3, DHRS4; N-acetylneuraminate lyase-like, NPL; RAC serine/threonine-protein kinase, AKT; phosphoglycerate mutase 1, PGAM; delta(3,5)-Delta(2,4)-dienoyl-CoA isomerase, mitochondrial, ECH1; glucose-6-phosphatase 3, G6PC; 1,4-alpha-glucan-branching enzyme, GBE1, glgB; ATP-dependent 6-phosphofructokinase, PFK; phosphoglycolate phosphatase 1A, chloroplastic-like, FPPP; galactokinase-like, galK; acidic mammalian chitinase-like, E3.2.1.14; DNA polymerase epsilon subunit 3, POLE3; U3 small nucleolar ribonucleoprotein protein IMP4, IMP4; HEAT repeat-containing protein 1 homolog, UTP10; exonuclease DPD1, TREX1; ribonuclease P protein subunit p25-like protein, RPP25; replication protein A 32 kDa subunit, RFA2;adenylate kinase isoenzyme 6 homolog, AK6; DNA repair protein complementing XP-C cells homolog, XPC;DNA-directed RNA polymerase I subunit RPA12, RPA12, POLR1H, ZNRD1; DNA-directed RNA polymerase II subunit Rpb4, RPB4, POLR2D; nucleolar GTP-binding protein 1, transcript variant X1, NOG1; enolase-like, transcript variant X2, ENO; U6 snRNA-associated Sm-like protein LSm6, LSM6; DNA repair endonuclease XPF, ERCC4, XPF; stimulator of interferon genes protein-like, TMEM173, MITA; probable RNA 3'-terminal phosphate cyclase-like protein, RCL1; general transcription factor IIH subunit 1, TFIIH1, GTF2H1, TFB1; DNA polymerase delta subunit 3, POLD3; U3 small nucleolar RNA-associated protein 15 homolog, UTP15; WD repeat-containing protein 36, UTP21, WDR36; serine/threonine-protein kinase RIO2, RIOK2; DNA-directed RNA polymerases I and III subunit RPAC1, RPAC1, RPC40, POLR1C; ribonuclease P/MRP protein subunit POP5, transcript variant X1, POP5; DNA-directed RNA polymerase I subunit RPA49-like, RPA49, POLR1E; ribosomal RNA small subunit methyltransferase NEP1, EMG1, NEP1; M-phase phosphoprotein 6, MPHOSPH6, MPP6; ribosome biogenesis protein BMS1 homolog, BMS1; U3 small nucleolar ribonucleoprotein protein IMP3, IMP3.
