# Supplementary material for: Insights from the transcriptome and metabolome into the molecular basis of diapause in Leguminivora glycinivorella (Lepidoptera, Olethreutidae)
Source: PLoS One. 2025 Jun 4;20(6):e0322332. doi: 10.1371/journal.pone.0322332 (PMC12136294; doi:10.1371/journal.pone.0322332)
Supplement: S9 Table — (DOCX) [file pone.0322332.s012.docx]

**Supporting Information S9 Table.** General Metabolite Profiles between the diapause and pre-diapause of *L.glycinivorella*.

| Superclass | Number |
| --- | --- |
| Lipids and lipid-like molecules | 395 |
| Organic acids and derivatives | 218 |
| Organoheterocyclic compounds | 412 |
| Phenylpropanoids and polyketides | 136 |
| Benzenoids | 134 |
| Organic oxygen compounds | 317 |
| Alkaloids and derivatives | 6 |
| Lignans, neolignans and related compounds | 4 |
| Hydrocarbons | 2 |
| Organic Polymers | 1 |
| Organic 1,3-dipolar compounds | 1 |
| Hydrocarbon derivatives | 1 |
| Homogeneous non-metal compounds | 1 |
