# Supplementary material for: Insights from the transcriptome and metabolome into the molecular basis of diapause in Leguminivora glycinivorella (Lepidoptera, Olethreutidae)
Source: PLoS One. 2025 Jun 4;20(6):e0322332. doi: 10.1371/journal.pone.0322332 (PMC12136294; doi:10.1371/journal.pone.0322332)
Supplement: S10 Table — (DOCX) [file pone.0322332.s013.docx]

**Supporting Information S10 Table.** Hierarchical cluster metabolites between the diapause and pre-diapause of *L.glycinivorella*.

| Metabolite | D_1 | D_2 | D_3 | PD_1 | PD_2 | PD_3 |
| --- | --- | --- | --- | --- | --- | --- |
| Adenosine | 5.766 | 5.731 | 5.751 | 3.714 | 3.716 | 3.709 |
| 5-Hydroxy-L-tryptophan | 4.221 | 4.398 | 4.107 | 2.474 | 2.476 | 2.623 |
| Sphingosine | 6.142 | 6.15 | 6.158 | 4.072 | 3.838 | 3.633 |
| PE(15:0/22:4(7Z,10Z,13Z,16Z)) | 5.814 | 5.935 | 5.89 | 4.155 | 4.14 | 4.497 |
| Glycerophosphocholine | 7.814 | 7.822 | 7.852 | 7.067 | 7.094 | 7.062 |
| Kynurenic Acid | 4.783 | 4.777 | 4.784 | 3.888 | 3.937 | 3.877 |
| N-Acetyl-1-aspartylglutamic acid | 4.643 | 4.639 | 4.679 | 2.867 | 2.858 | 2.852 |
| 4-Hydroxyphenylpyruvic acid | 5.565 | 5.594 | 5.505 | 2.397 | 2.388 | 2.382 |
| Pantothenic Acid | 6.846 | 6.94 | 6.935 | 6.054 | 6.017 | 6.02 |
| Biotin | 5.544 | 5.539 | 5.543 | 4.812 | 4.75 | 4.788 |
| N6-(1,2-Dicarboxyethyl)-AMP | 6.444 | 6.41 | 6.403 | 7.226 | 7.229 | 7.227 |
| Citric acid | 5.378 | 5.387 | 5.389 | 6.122 | 6.122 | 6.135 |
| PA(8:0/a-15:0) | 2.18 | 2.177 | 2.172 | 5.185 | 5.158 | 5.085 |
| 9(S)-HODE | 3.062 | 3.06 | 3.055 | 4.934 | 4.947 | 4.931 |
| Glutaric acid | 4.607 | 4.566 | 4.498 | 5.43 | 5.442 | 5.44 |
| 3-Hydroxy-N6,N6,N6-trimethyl-L-lysine | 3.984 | 3.766 | 3.898 | 4.5 | 4.511 | 4.585 |
| N-acetylaspartate | 4.556 | 4.566 | 4.579 | 5.508 | 5.492 | 5.509 |
| Adenylsuccinic acid | 4.222 | 3.679 | 3.675 | 5.353 | 5.33 | 5.353 |
| Pyruvic Acid | 3.047 | 3.274 | 3.055 | 4.501 | 4.517 | 4.502 |
| Adenylosuccinate | 6.518 | 6.505 | 6.505 | 7.303 | 7.3 | 7.3 |
