# Supplementary material for: Insights from the transcriptome and metabolome into the molecular basis of diapause in Leguminivora glycinivorella (Lepidoptera, Olethreutidae)
Source: PLoS One. 2025 Jun 4;20(6):e0322332. doi: 10.1371/journal.pone.0322332 (PMC12136294; doi:10.1371/journal.pone.0322332)
Supplement: S11 Table — (DOCX) [file pone.0322332.s014.docx]

**Supporting Information S11 Table.** The top10 enriched KEGG pathway of the DAMs between he diapause and pre-diapause of *L.glycinivorella*.

|  | number | First Category | Pathway Desciption | Pathway_ID | P_value |
| --- | --- | --- | --- | --- | --- |
| up-regulated | 16 | Metabolism | Tryptophan metabolism | map00380 | 8.71E-07 |
|  | 8 | Metabolism | Tyrosine metabolism | map00350 | 0.002255 |
|  | 6 | Environmental Information Processing | Neuroactive ligand-receptor interaction | map04080 | 0.004463 |
|  | 8 | Metabolism | Drug metabolism - cytochrome P450 | map00982 | 0.004474 |
|  | 8 | Metabolism | Glycerophospholipid metabolism | map00564 | 0.00645 |
|  | 3 | Environmental Information Processing | Sphingolipid signaling pathway | map04071 | 0.009451 |
|  | 5 | Metabolism | Phenylalanine, tyrosine and tryptophan biosynthesis | map00400 | 0.01842 |
|  | 3 | Metabolism | Caffeine metabolism | map00232 | 0.02742 |
|  | 4 | Organismal Systems | Vitamin digestion and absorption | map04977 | 0.02911 |
|  | 3 | Environmental Information Processing | cAMP signaling pathway | map04024 | 0.03836 |
|  | 4 | Metabolism | alpha-Linolenic acid metabolism | map00592 | 0.04283 |
| down-regulated | 7 | Metabolism | Linoleic acid metabolism | map00591 | 6.16E-05 |
|  | 6 | Metabolism | Lysine degradation | map00310 | 0.001636 |
|  | 4 | Metabolism | Valine, leucine and isoleucine biosynthesis | map00290 | 0.002555 |
|  | 8 | Organismal Systems | Fat digestion and absorption | map04975 | 0.00397 |
|  | 4 | Metabolism | Cutin, suberine and wax biosynthesis | map00073 | 0.004678 |
|  | 7 | Metabolism | Alanine, aspartate and glutamate metabolism | map00250 | 0.00535 |
|  | 11 | Metabolism | Glycerophospholipid metabolism | map00564 | 0.01409 |
|  | 5 | Metabolism | alpha-Linolenic acid metabolism | map00592 | 0.02589 |
|  | 5 | Organismal Systems | Dopaminergic synapse | map04728 | 0.03684 |
|  | 5 | Metabolism | Tyrosine metabolism | map00350 | 0.04996 |
