# Supplementary material for: Insights from the transcriptome and metabolome into the molecular basis of diapause in Leguminivora glycinivorella (Lepidoptera, Olethreutidae)
Source: PLoS One. 2025 Jun 4;20(6):e0322332. doi: 10.1371/journal.pone.0322332 (PMC12136294; doi:10.1371/journal.pone.0322332)
Supplement: S12 Table — (DOCX) [file pone.0322332.s015.docx]

**Supporting Information S12 Table.** The top 20 KEGG pathways enriched in DEGs and DAMs between the diapause and pre-diapause of *L.glycinivorella*.

| Pathway ID | Pathway Description | First Category | Database | Metabolite number | Pvalue_ metabolite | Gene number | Pvalue_ gene |
| --- | --- | --- | --- | --- | --- | --- | --- |
| map00190 | Oxidative phosphorylation | Metabolism | KEGG PATHWAY | 1 | 0.2848216 | 87 | 1.95E-13 |
| map00280 | Valine, leucine and isoleucine degradation | Metabolism | KEGG PATHWAY | 1 | 0.58627407 | 38 | 2.06E-12 |
| map05208 | Chemical carcinogenesis - reactive oxygen species | Human Diseases | KEGG PATHWAY | 1 | 0.69874233 | 111 | 4.59E-12 |
| map00010 | Glycolysis / Gluconeogenesis | Metabolism | KEGG PATHWAY | 0 | - | 39 | 3.09E-11 |
| map00071 | Fatty acid degradation | Metabolism | KEGG PATHWAY | 0 | - | 32 | 1.71E-10 |
| map05415 | Diabetic cardiomyopathy | Human Diseases | KEGG PATHWAY | 3 | 0.04589632 | 97 | 2.68E-10 |
| map00410 | beta-Alanine metabolism | Metabolism | KEGG PATHWAY | 1 | 0.48913978 | 31 | 1.30E-08 |
| map00640 | Propanoate metabolism | Metabolism | KEGG PATHWAY | 0 | - | 24 | 2.61E-08 |
| map04932 | Non-alcoholic fatty liver disease | Human Diseases | KEGG PATHWAY | 0 | - | 71 | 2.67E-07 |
| map04974 | Protein digestion and absorption | Organismal Systems | KEGG PATHWAY | 0 | - | 69 | 7.62E-07 |
| map00380 | Tryptophan metabolism | Metabolism | KEGG PATHWAY | 10 | 4.40E-05 | 27 | 9.50E-07 |
| map04714 | Thermogenesis | Organismal Systems | KEGG PATHWAY | 0 | - | 95 | 1.94E-06 |
| map00020 | Citrate cycle (TCA cycle) | Metabolism | KEGG PATHWAY | 1 | 0.34243931 | 26 | 2.13E-06 |
| map00330 | Arginine and proline metabolism | Metabolism | KEGG PATHWAY | 1 | 0.76645047 | 26 | 4.68E-06 |
| map00270 | Cysteine and methionine metabolism | Metabolism | KEGG PATHWAY | 1 | 0.75108724 | 25 | 4.72E-06 |
| map00260 | Glycine, serine and threonine metabolism | Metabolism | KEGG PATHWAY | 1 | 0.63553211 | 26 | 9.76E-06 |
| map04972 | Pancreatic secretion | Organismal Systems | KEGG PATHWAY | 0 | - | 69 | 1.16E-05 |
| map05012 | Parkinson disease | Human Diseases | KEGG PATHWAY | 3 | 0.01581686 | 108 | 1.62E-05 |
| map00980 | Metabolism of xenobiotics by cytochrome P450 | Metabolism | KEGG PATHWAY | 2 | 0.72102024 | 40 | 2.06E-05 |
| map05204 | Chemical carcinogenesis - DNA adducts | Human Diseases | KEGG PATHWAY | 1 | 0.80298267 | 39 | 2.35E-05 |

Note: P<0.05 is represented by *, P<0.01 is represented by **.
