# Supplementary material for: Insights from the transcriptome and metabolome into the molecular basis of diapause in Leguminivora glycinivorella (Lepidoptera, Olethreutidae)
Source: PLoS One. 2025 Jun 4;20(6):e0322332. doi: 10.1371/journal.pone.0322332 (PMC12136294; doi:10.1371/journal.pone.0322332)
Supplement: Attachment — Submitted filename: comments_PONE-D-24-57514_25.01.31.docx [file pone.0322332.s016.docx]

The authors presented a study aimed at identifying the key substances of diapause regulation by differential expression genes and differential metabolites, using transcriptome and metabolomics in the diapause and pre-diapause stages in L. glycinivorella. They found that metabolic regulation involving TCA cycle, glycolysis, insulin signaling pathway, and glycerophospholipid metabolic pathway, which suggesting that they may be closely related to energy reserve, immune regulation and hormone regulation during diapause. I have a hard time finding key results related to the described discussion… Therefore, I recommend that some revisions be made and specific questions be addressed before the paper is published.

[Abstract]

- In the abstract, insulin signaling is presented as a major pathway; however, there is a lack of related discussion in the results and discussion sections.

[Introduction]

- It is necessary to present a concise and clear explanation of diapause in the introduction, focusing on its relevance to the study.
- It would be beneficial to present the advantages and necessity of integrating transcriptomics and metabolomics in molecular mechanism studies.

[Method]

- It is necessary to specify the number of samples used in the transcriptomics and metabolomics analyses.
- I can’t determine the statistical analysis been performed appropriately and rigorously. It is necessary to specify the data analysis methods, statistical method, and the statistical software used in the transcriptome and metabolome analysis.
- The sample preparation method for metabolomics, as well as the LC-MS analysis procedure, should be described.
- It is necessary to present information on the identified metabolites in a supplementary table with retention time, m/z, ppm error, CV%, intensity or fold change between diapause and pre-diapause stages, p-value.

[Results]

- 9(S)-HODE and PA(8:0/a-15:0) are not commonly observed under metabolite and lipid profiling conditions. Have their peaks been confirmed, and have they been validated using standard compounds?
- In Figure S4, the colors and shapes of PCR and RNA-seq data are too similar, making them difficult to distinguish. It is necessary to represent them differently.
- The content of the following sentence does not match the figure legend, and the labels "A" and "B" are missing from Figure 3. Revisions are needed.

“50 of 107 were higher in PD (Figure 3B), and the top 5 up-regulated metabolites were Adenylosuccinate, 9(S)-HODE, PA(8:0/a15:0), N6-(1,2-Dicarboxyethyl)-AMP, and N-acetylaspartate. 57 of 107 were higher in D, and the top 5 down-regulated metabolites were 5-Hydroxy-L-tryptophan, PE (15:0/22:4(7Z,10Z,13Z,16Z)), Sphingosine, Pantothenic Acid, and Glycerophosphocholine.”

- It is difficult to find the level changes of DEGs in the paper through figures or tables. It is necessary to present the data more clearly for easier reference.

“These results showed that, in the developmental process from PD to D, most DEGs were related to the immune defense, energy metabolism and hormone synthesis. There were three down-regulated DEGs related to insect immunity and defense (heat shock protein genes (HSP), Superoxide Dismutase (SOD), and catalase(CAT)), six down regulated DEGs related to energy metabolism (hexokinase (HK), pyruvate kinase (PK), ATP-dependent 6-phosphofructokinase (PFK), citrate synthase (CS), isocitrate dehydrogenase [NADP] cytoplasmic (IDH), α,α-trehalose-phosphate synthase [UDP forming] (TPS)), and two down-regulated DEGs related to hormone synthesis (juvenile hormone epoxide hydrolase (JHEH) and 20-hydroxyecdysone (20E)).”

“In this study, it was found that JHEH was down-regulated during diapause, thereby inhibiting the breakdown of juvenile hormones and keeping the larvae in the larval state for a long time, which may be the reason why SPB maintains diapause with mature larvae.”

[Discussion]

- Please provide the evidence based on the results to support the conclusion stated in the sentence on page 17, line 253 of the Discussion section.

“it is of great significance to study the transcriptome and metabolome of soybean insect at different diapause stages.”

- If there are existing studies related to the following sentence, relevant references should be added.

“Certain metabolites, such as Phosphatidyl choline (PC) and proline, can act as cryoprotectants to improve cold tolerance in diapausing insects.”

- Figure 4 appears to represent the key metabolic pathways identified in this study. Therefore, it is necessary to provide an interpretation and discussion of the key metabolites and transcriptomic changes observed in the study based on the measured results. Including unverified metabolites and transcripts in the interpretation may lead to overstatements. For example, regarding glycerol aldehyde 3-phosphate (G3P), the discussion includes changes in glycerol kinase (GK), glucose, glycogen, and glycerol. It is important to specify whether changes in glycerol kinase (GK), glucose, glycogen, and glycerol were actually observed.
- The title "4.2 Immune Response in the Diapause" primarily refers to immune response; however, the content mainly discusses ROS and antioxidants. A more appropriate revision of the title is needed to accurately reflect the content.

[Minor]

- Spelling corrections are needed throughout various sections of the paper.
