# Supplementary material for: Insights from the transcriptome and metabolome into the molecular basis of diapause in Leguminivora glycinivorella (Lepidoptera, Olethreutidae)
Source: PLoS One. 2025 Jun 4;20(6):e0322332. doi: 10.1371/journal.pone.0322332 (PMC12136294; doi:10.1371/journal.pone.0322332)
Supplement: Attachment — Submitted filename: renamed_4dc79.docx [file pone.0322332.s018.docx]

**Responses to the reviewer’s comments**

**(Manuscript ID: PONE-D-24-57514)**

Note: our responses to the reviewers are written in blue font, and changes to the manuscript are shown in red font. Line and page numbers refer to the “changes in red” version of the manuscript.

**Reviewer1：**

The authors presented a study aimed at identifying the key substances of diapause regulation by differential expression genes and differential metabolites, using transcriptome and metabolomics in the diapause and pre-diapause stages in L. glycinivorella. They found that metabolic regulation involving TCA cycle, glycolysis, insulin signaling pathway, and glycerophospholipid metabolic pathway, which suggesting that they may be closely related to energy reserve, immune regulation and hormone regulation during diapause. I have a hard time finding key results related to the described discussion… Therefore, I recommend that some revisions be made and specific questions be addressed before the paper is published.

We also appreciate your clear and detailed feedback and hope that the explanation has fully addressed all of your concerns. In the remainder of this letter, we discuss each of your comments individually along with our corresponding responses.

1. [Abstract]：In the abstract, insulin signaling is presented as a major pathway; however, there is a lack of related discussion in the results and discussion sections.

Thanks for your suggestions. In the abstract, we believe that insulin signaling is presented as a major pathway, and we have made corresponding additions to this content in the results. Insulin signaling pathway was significantly enriched in transcriptome KEGG enrichment analysis (P<0.01), and we believe that insulin signaling pathway may be a major participant in diapause metabolite regulation, so this part of the content is supplemented as described below. In the discussion section, we discuss the serine/threonine protein kinase (AKT) along this pathway, as well as the related enzyme GYS, which may influence glycogen synthesis and metabolism.

Page 11, lines 241~252:

KEGG pathways of DEGs could give deeper insights into the physiological processes related to diapause. Of all gene with a KEGG annotation, 2262 up-regulated DEGs were assigned to 318 KEGG pathways, and 5065 down-regulated DEGs were assigned to 336 KEGG pathways. Up-regulated DEGs were classified into “Ribosome biogenesis in eukaryotes”, “RNA degradation”, “Nucleotide excision repair”, and “RNA polymerase” (Figure 1C). Down-regulated DEGs were classified into “Glycolysis/Gluconeogenesis”, “Amino sugar and nucleotide sugar metabolism”, “Peroxisome”, and “Tryptophan metabolism” (Figure 1D). Top 10 KEGG pathways were significantly enriched, are listed in Table S7. In addition, some metabolic pathways are also significantly enriched, such as insulin signaling pathway, which may be a major participant in the regulation of metabolites in diaphaesis (32), in which serine/threonine protein kinase (AKT) is involved.

Page 18, lines 402~423:

Glucose can also be converted into glycerol, which can be used as an anti-freezing protective agent to enhance the cold tolerance of insects (37). Glycerol kinase (GK) is a rate-limiting enzyme in glycerolipid metabolism by the conversion of glycerol aldehyde 3-phosphate (G3P) to phospholipids such as glyceryl phosphate. The catabolism of glycerol in vivo consists of a two-step reaction, in which glycerol is catalyzed by glycerol kinase and phosphorylated to produce glycerol 3-phosphate. Rapid cold acclimation of Sarcophaga crassipalpis and Spodoptera exigua showed that they acted as the main anti-freeze protective substances by increasing their glycerol content (47). During diapause, GK was significantly down-regulated, This indicates the accumulation of glycerol in SPB, suggesting that this enzyme is mainly used as a protective substance to enhance the cold resistance of insects to survive diapause environment. As a key core factor in insulin signaling pathway, PDK1 phosphorylates and activates serine/threonine protein kinase (AKT), thereby regulating glucose homeostasis (48). An important gene downstream of AKT is glycogen synthase kinase 3β (GSK3β), which is involved in glycogen biosynthesis and metabolism. GSK3β inhibits glycogen synthase by phosphorylating glycogen synthase (GYS). In this way, it inhibits glycogen biosynthesis (49). In this study, by inhibiting GSK3β, AKT was able to overcome this inhibition and increase GYS activity, thereby affecting glycogen content.In this study, no significant differences in metabolites such as glycogen, glucose and glycerol were detected, but significant changes in transcription factors were found in this process, conjecturing that the TCA cycle and glycolysis process are dynamic changes, The entire metabolic network is in homeostasis.

2. [Introduction]：It is necessary to present a concise and clear explanation of diapause in the introduction, focusing on its relevance to the study.

Thanks for your suggestions. On the basis of the original manuscript, we have added a concise explanation of diapause and deleted the characteristics of diapause, and finally added the correlation between diapause and the article. We have provided detailed explanations of the changes made in the revised manuscript, including the specific locations where these changes can be found.

Page 3, lines 58~81:

Diapause is defined as a period of suspended development in insects and other invertebrates during unfavorable environmental conditions (7). In insects, diapause is a means of avoiding mortality and adjusting to the harsh surroundings (8). Metabolic suppression is a diapause mark that enables insects to ~~align arrange~~ synchronize their life cycles to coincide with times that are suitable for development, growth, and reproduction and survive unfavorable seasons (9). Diapause is a developmental stop state that is genetically determined and can happen at the embryonic stage (10), larval stage (11), pupal stage (12) and adult stage (13). ~~And the types of diapause~~ Diapause types are divided into obligate and facultative according to whether it can be influenced by environmental factors (14). Insect diapause is a dynamic process successive phases, which includes three periods: pre-diapause, diapause and post-diapause. In the stage of pre-diapause, SPB stores food, changes behavior and reduces development when the stimuli reach some critical level (15). ~~Diapause slows or even prevents SPB growth and development, mostly through decreased activity and feeding intake, as well as the inability to finish the pupate and eclosion stages of development.~~ Insect diapause is characterized by changes in the body, primarily a decreased metabolic rate and an improved tolerance to temperature and moisture (16,17). When the conditions change to be suitable for SPB, the insects recover from diapause with the metabolic and the physiological level returning to normal (6). SPB is a typical ~~univolitine~~ univoltine insect with obligatory diapause, and the larvae enter the pods to feed and develop until they are fourth instar larvae. They leave the pods, and overwinter in cocoons in the soil layer of 3~~~~~ – 15 cm (18–20). In order to achieve the goal of controlling the reproduction of this pest in a more environmentally friendly way, it is necessary to understand the molecular mechanism of diapause in SPB.

3. It would be beneficial to present the advantages and necessity of integrating transcriptomics and metabolomics in molecular mechanism studies.

Thanks for your suggestions. The advantages and necessity of combining transcriptomics and metabolomics in the study of molecular mechanisms have been added to the original manuscript, as described below.

Page 5, lines 101~113:

Metabolomics is the subject of qualitative and quantitative analysis of small molecules simultaneously, mainly studying the exposure of living organisms Changes of metabolites in vivo after boundary stimulation may change regularly with time (15). Transcriptomics (RNA-Seq) is an important method to study gene function and structure. Studying various genes in individuals, tissues or cells under different conditions is an important aspect of genomics, which has short sequencing time, large sequencing capacity and low sequencing cost(16,17). The combination of the transcriptome and metabolome better reflects the environment of the cell. The aims of this research are analyzing differentially expressed genes and metabolites and their functions of SPB in diapause and pre-diapause period with RNA-seq technology and targeted liquid chromatography mass spectrometry (LC-MS), ~~and the molecular mechanism of diapause will be revealed~~ and to elucidate the molecular mechanisms underlying diapause.

4. [Method]：It is necessary to specify the number of samples used in the transcriptomics and metabolomics analyses.

We were really sorry for our careless mistakes. Thank you for your reminder. The number of samples for transcriptomic and metabolomic analysis has been added to the manuscript.

Page 6, lines 116~123:

All samples were collected from the soybean fields in Xing’an League, Inner Mongolia (122°52'E, 46°49'N) on September, 2023. The forth instar larvae, pre-diapause (PD, were white and still feeding) and diapause (D, were red and had stopped feeding and developing for about 30d, Figure S1), were stored in -80°C. ~~Each sample was divided into two equal parts for the transcriptomics and metabolomics.~~ A total of 24 samples from 2 periods were collected, and each sample was divided into two equal parts for transcriptomics and metabolomics. (Three replicates per period for the transcriptome and nine replicates per period for the metabolome).

5. I can’t determine the statistical analysis been performed appropriately and rigorously. It is necessary to specify the data analysis methods, statistical method, and the statistical software used in the transcriptome and metabolome analysis.

Thanks for your suggestions, For strict statistical analysis, data analysis methods, statistical methods, and statistical software used in transcriptome and metabolome analysis are specified in the manuscript and marked in red for easy observation.

Page 6, lines 125~205:

Total RNA was isolated from the tissue specimens employing TRIzol® Reagent in strict adherence to the supplier's protocol. Subsequently, RNA integrity assessment was conducted using an Agilent 5300 Bioanalyser system, followed by spectrophotometric quantification with a NanoDrop ND-2000 instrument. At Shanghai Majorbio Bio-pharm Biotechnology Co., Ltd. (Shanghai, China), the RNA purification, reverse transcription, library building, and sequencing procedures were completed. ~~To create the SPB RNA-seq transcriptome library, 1μg of total RNA and Illumina® Stranded mRNA Prep, Ligation (San Diego, CA) were used. The NovaSeq X Plus platform (PE150) was used to perform the sequencing library after Qubit 4.0 quantification, using the NovaSeq Reagent Kit (NovaSeq6000).~~The workflow comprised: PolyA+ mRNA enrichment via oligo(dT) magnetic bead selection followed by chemical fragmentation; First-strand cDNA synthesis with random hexamer primers using SuperScript™ ds-cDNA Synthesis Kit (Invitrogen, CA); Second-strand synthesis, end-repair/A-tailing, and Illumina adapter ligation per standardized workflows; Size selection (~300 bp fragments) through 2% Low Range Ultra Agarose electrophoresis; PCR amplification (15 cycles) with Phusion High-Fidelity DNA Polymerase (NEB, USA); Library quantification via Qubit® 4.0 Fluorometer prior to paired-end sequencing (PE150) on NovaSeq 6000 Plus platform with NovaSeq Reagent Kits. Clean reads in orientation mode were individually aligned to the reference genome using HISAT2 software(27). http://www.ncbi.nlm.nih.gov/datasets/genome/GCF_023078275.1/ is the website from which we obtained the SPB reference genome. Using a reference-based process, StringTie put together each sample's mapped reads(28). Each transcript's expression level was determined using the transcripts per million reads (TPM) method. The analysis of differential expression was done with DESeq2(29). Genes with FDR="0.05" and |log2FC|≧1 were identified as substantially divergent expressed genes (DEGs)(30). GO and KEGG functional-enrichment analysis were carried out by Goatools and Python scipy software, respectively, were performed to identify DEGs that were significantly enriched in GO and metabolic pathways.

**2.3 Extraction and analysis of Metabolite**

50 mg SPB were transferred into 2 mL cryotubes preloaded with 6 mm stainless steel grinding beads. Metabolite extraction was initiated by adding 400 μL of ice-cold methanol:water (4:1, *v*/*v*) solution containing 0.02 mg/mL L-2-chlorophenylalanine (internal standard). Mechanical homogenization was performed using a Wonbio-96c cryogenic grinder (Shanghai Wanbo Biotechnology Co., Ltd.) at 50 Hz for 6 minutes under -10°C conditions, immediately succeeded by ultrasonication at 40 kHz for 30 minutes in a temperature-controlled chamber (5°C). Following this, the homogenates underwent: ① Cryopreservation at -20°C for 30 minutes to enhance macromolecule precipitation; ② Phase separation via centrifugation at 13,000 × g for 15 minutes (4°C); ③ Precise supernatant collection using calibrated micropipettes; ④ Final filtration through 0.22 μm into LC-MS certified vials prior to instrumental analysis.

~~Using an ACQUITY HSS T3 column (100 mm × 2.1 mm i.d., 1.8 μm; Waters, USA),~~ The SPBs were subjected to LC-MS/MS analysis at Majorbio Bio-Pharm Technology Co. Ltd. (Shanghai, China) using a UHPLC-Q Exactive HF-X system, equipped with an ACQUITY HSS T3 column (100 mm × 2.1 mm i.d., 1.8 μm; Waters, USA). The mobile phases consisted of 0.1% formic acid in water:acetonitrile (95:5, v/v) (solvent A) and 0.1% formic acid in acetonitrile:isopropanol:water (47.5:47.5, v/v) (solvent B). The flow rate was 0.40 mL/min and the column temperature was 40℃. The injection volume was 3 μL.

Thermo UHPLC-Q Exactive HF-X Mass Spectrometer was the mass spectrometer used for data collection. There are two possible modes of operation for its electrospray ionization (ESI) source. The conditions were set as followed: Aux gas heating temperature at 425℃; Capillary temp at 325℃; sheath gas flow rate at 50 psi; Aux gas flow rate at 13 psi; ion-spray voltage floating (ISVF) at -3500V in negative mode and 3500V in positive mode, respectively; Normalized collision energy , 20-40-60 eV rolling for MS/MS. Full MS resolution was 60000, and MS/MS resolution was 7500. Data acquisition was performed with the Data Dependent Acquisition (DDA) mode. The detection was carried out over a mass range of 70-1050 m/z. A uniform format was created from the UHPLC-MS raw data using Progenesis QI software (Waters, Milford, USA) through baseline filtering, peak identification, peak integral, retention time correction, and peak alignment. Then, the data matrix containing sample names, m/z, retention time and peak intensities was exported for further analyses (TableS1). Majorbio Biotechnology Co., Ltd. (Shanghai, China) self-compiled the Majorbio Database (MJDB), the HMDB (http://www.hmdb.ca/), and Metlin (https://metlin.scripps.edu/) as the primary databases for metabolite identification. Using the R package "ropls" (Version 1.6.2), the discriminant principle component analysis (PCA), least partial squares discriminant analysis (PLS-DA), and a seven-cycle interactive validation were performed(31). They evaluated the model's stability. Version 1.6.2 of the PLS-DA model's variable importance projection (VIP) was employed to check for differentially accumulating metabolites(32). Differentially accumulated metabolites (DAMs) were defined as those in which the VIP was greater than 1 and the Fold Change was greater than 2 or less than 0.5. Two groups' distinct metabolites were mapped into their corresponding biochemical pathways using metabolic enrichment and pathway analysis based on the KEGG database (<http://www.genome.jp/kegg/>).

**2.4 Combined Analysis of the Transcriptome and Metabolome of SPB**

Using internet resources, metabolome and transcriptome data were integrated using OmicsPLS (R packages) software for bidirectional orthogonal projections to latent structures (O2PLS). We were able to extract information about shared pathways for both datasets by connecting DEGs and DAMs to the KEGG pathway database (scipy(Python)).

6. The sample preparation method for metabolomics, as well as the LC-MS analysis procedure, should be described.

We sincerely thank the reviewer for careful reading. We are sorry for our carelessness. We have added to The revised draft, the sample preparation method for metabolomics, as well as the LC-MS analysis procedure.

Page 8, lines 155~199:

50 mg SPB were transferred into 2 mL cryotubes preloaded with 6 mm stainless steel grinding beads. Metabolite extraction was initiated by adding 400 μL of ice-cold methanol:water (4:1, *v*/*v*) solution containing 0.02 mg/mL L-2-chlorophenylalanine (internal standard). Mechanical homogenization was performed using a Wonbio-96c cryogenic grinder (Shanghai Wanbo Biotechnology Co., Ltd.) at 50 Hz for 6 minutes under -10°C conditions, immediately succeeded by ultrasonication at 40 kHz for 30 minutes in a temperature-controlled chamber (5°C). Following this, the homogenates underwent: ① Cryopreservation at -20°C for 30 minutes to enhance macromolecule precipitation; ② Phase separation via centrifugation at 13,000 × g for 15 minutes (4°C); ③ Precise supernatant collection using calibrated micropipettes; ④ Final filtration through 0.22 μm into LC-MS certified vials prior to instrumental analysis.

~~Using an ACQUITY HSS T3 column (100 mm × 2.1 mm i.d., 1.8 μm; Waters, USA),~~ The SPBs were subjected to LC-MS/MS analysis at Majorbio Bio-Pharm Technology Co. Ltd. (Shanghai, China) using a UHPLC-Q Exactive HF-X system, equipped with an ACQUITY HSS T3 column (100 mm × 2.1 mm i.d., 1.8 μm; Waters, USA). The mobile phases consisted of 0.1% formic acid in water:acetonitrile (95:5, v/v) (solvent A) and 0.1% formic acid in acetonitrile: isopropanol: water (47.5:47.5, v/v) (solvent B). The flow rate was 0.40 mL/min and the column temperature was 40℃. The injection volume was 3 μL.

Thermo UHPLC-Q Exactive HF-X Mass Spectrometer was the mass spectrometer used for data collection. There are two possible modes of operation for its electrospray ionization (ESI) source. The conditions were set as followed: Aux gas heating temperature at 425℃; Capillary temp at 325℃; sheath gas flow rate at 50 psi; Aux gas flow rate at 13 psi; ion-spray voltage floating (ISVF) at -3500V in negative mode and 3500V in positive mode, respectively; Normalized collision energy , 20-40-60 eV rolling for MS/MS. Full MS resolution was 60000, and MS/MS resolution was 7500. Data acquisition was performed with the Data Dependent Acquisition (DDA) mode. The detection was carried out over a mass range of 70-1050 m/z. A uniform format was created from the UHPLC-MS raw data using Progenesis QI software (Waters, Milford, USA) through baseline filtering, peak identification, peak integral, retention time correction, and peak alignment. Then, the data matrix containing sample names, m/z, retention time and peak intensities was exported for further analyses(TableS1). Majorbio Biotechnology Co., Ltd. (Shanghai, China) self-compiled the Majorbio Database (MJDB), the HMDB (http://www.hmdb.ca/), and Metlin (https://metlin.scripps.edu/) as the primary databases for metabolite identification. Using the R package "ropls" (Version 1.6.2), the discriminant principle component analysis (PCA), least partial squares discriminant analysis (PLS-DA), and a seven-cycle interactive validation were performed(18). They evaluated the model's stability. Version 1.6.2 of the PLS-DA model's variable importance projection (VIP) was employed to check for differentially accumulating metabolites(19). Differentially accumulated metabolites (DAMs) were defined as those in which the VIP was greater than 1 and the Fold Change was greater than 2 or less than 0.5. Two groups' distinct metabolites were mapped into their corresponding biochemical pathways using metabolic enrichment and pathway analysis based on the KEGG database (<http://www.genome.jp/kegg/>).

7. It is necessary to present information on the identified metabolites in a supplementary table with retention time, m/z, ppm error, CV%, intensity or fold change between diapause and pre-diapause stages, p-value.

Thanks for your suggestions. This section was revised and modified according to the information showed in the work suggested by the reviewer. We have added relevant information in the supplement table, including metabolite class, retention time, m/z, ppm error, CV%, P-value for reference.

Supporting Information Table S1.

Table S1 Identification information of metabolites of *L.glycinivorella*.

| Serial number | Metabolite | M/Z | Intensity | Retention time | CV% | ppm Error | Fold Change | p-value |
| --- | --- | --- | --- | --- | --- | --- | --- | --- |
| Amino acids and their derivatives | | | | | | | | |
| 1 | 2-Aminobenzoic Acid | 120.04 | 4.4 | 2.43 | 11.58 | 0.85 | 1.24 | ** |
| 2 | 2-Keto-6-acetamidocaproate | 229.12 | 3.67 | 3.19 | 22.68 | 1.77 | 0.64 | ** |
| 3 | 3-Hydroxy-N6,N6,N6-trimethyl-L-lysine | 205.15 | 4.25 | 0.57 | 8.32 | 3.55 | 0.86 | ** |
| 4 | 5-Hydroxy-L-tryptophan | 262.12 | 3.26 | 2.74 | 28.32 | 0.64 | 1.68 | ** |
| 5 | L-Aspartate-semialdehyde | 159.08 | 5.21 | 0.62 | 13.34 | 1.34 | 1.28 | ** |
| 6 | N-Acetyl-1-aspartylglutamic acid | 303.08 | 3.63 | 1.99 | 26.44 | 0.46 | 1.63 | ** |
| 7 | N-Acetyl-L-aspartic acid | 176.06 | 3.95 | 1.07 | 20.47 | 0.95 | 0.67 | ** |
| 8 | N6-Acetyl-L-lysine | 187.11 | 4.48 | 3.45 | 11.4 | 3.15 | 0.8 | ** |
| 9 | Saccharopine | 277.14 | 4.52 | 0.62 | 10.18 | 1.36 | 0.82 | ** |
| Nucleotides and their derivatives | | | | | | | | |
| 10 | Adenosine | 268.1 | 4.59 | 0.7 | 23.74 | 1.69 | 1.55 | ** |
| 11 | Adenylosuccinate | 462.07 | 6.96 | 2.71 | 6.08 | 0.53 | 0.89 | ** |
| 12 | Adenylsuccinic acid | 462.07 | 4.71 | 0.71 | 17.31 | 1.06 | 0.72 | * |
| 13 | CDP-ethanolamine | 445.05 | 4.89 | 0.63 | 8.73 | 1.4 | 0.85 | ** |
| Hormones and signaling molecules | | | | | | | | |
| 14 | Endomorphin-2 | 594.27 | 4.85 | 3.86 | 14.05 | 0.02 | 1.3 | ** |
| 15 | Norepinephrine | 214.07 | 3.99 | 3.69 | 24.86 | 2.31 | 1.58 | ** |
| 16 | Normetanephrine | 164.07 | 4.05 | 1.01 | 11.93 | 3.95 | 1.25 | ** |
| 17 | P-Octopamine | 152.07 | 3.79 | 1.35 | 20 | 5.18 | 1.45 | ** |
| Lipids (glycerolipids) | | | | | | | | |
| 18 | DG(15:0/18:3(6Z,9Z,12Z)/0:0) | 599.46 | 5.15 | 7.34 | 46.55 | 1.73 | 0.37 | * |
| 19 | DG(15:0/18:4(6Z,9Z,12Z,15Z)/0:0) | 597.45 | 4.65 | 7.18 | 43.41 | 1.55 | 0.4 | ** |
| 20 | LysoPC(20:0/0:0) | 596.37 | 4.7 | 6.18 | 27.03 | 4.04 | 0.58 | ** |
| 21 | LysoPC(22:4(7Z,10Z,13Z,16Z)/0:0) | 536.35 | 6.76 | 6.3 | 17.04 | 4.35 | 0.72 | ** |
| 22 | PA(8:0/a-13:0) | 517.29 | 3.78 | 6.23 | 59.14 | 0.28 | 0.25 | ** |
| 23 | PA(8:0/a-15:0) | 586.35 | 3.87 | 5.89 | 40.96 | 0.46 | 0.42 | ** |
| 24 | PC(22:4(7Z,10Z,13Z,16Z)/18:2(9Z,12Z)) | 816.59 | 5.13 | 6.69 | 14.8 | 3.63 | 0.76 | * |
| 25 | PE(15:0/22:4(7Z,10Z,13Z,16Z)) | 736.53 | 4.96 | 7.62 | 17.6 | 6.49 | 1.38 | ** |
| 26 | PE-NMe2(15:0/20:3(8Z,11Z,14Z)) | 800.54 | 5.93 | 6.5 | 10.5 | 0.57 | 1.21 | ** |
| 27 | PG(18:0/18:2(9Z,12Z)) | 816.57 | 4.86 | 6.76 | 18.34 | 1.61 | 1.4 | ** |
| 28 | PS(15:0/24:1(15Z)) | 830.59 | 4.5 | 6.51 | 17.08 | 0.7 | 1.31 | * |
| Lipids (sphingolipids) | | | | | | | | |
| 29 | SM(d18:1/12:0) | 669.49 | 4.55 | 7.62 | 21.61 | 1.55 | 1.48 | ** |
| 30 | Sphingosine | 300.29 | 4.83 | 6.09 | 25.59 | 1.49 | 1.6 | ** |
| Lipids (fatty acid derivatives) | | | | | | | | |
| 31 | 11-beta-Hydroxyandrosterone-3-glucuronide | 482.25 | 3.14 | 6.16 | 74.27 | 2.9 | 4.19 | * |
| 32 | 13(S)-HpODE | 311.22 | 4.69 | 6.85 | 23.68 | 0.19 | 0.63 | ** |
| 33 | 16-Hydroxyhexadecanoic acid | 290.27 | 5.21 | 6.06 | 9.68 | 1.82 | 0.83 | ** |
| 34 | 18-hydroxyoleate | 593.48 | 4.8 | 6.71 | 20.62 | 1.26 | 0.67 | ** |
| 35 | Alpha-Dimorphecolic acid | 279.23 | 4.47 | 7.51 | 15.22 | 1.27 | 0.75 | ** |
| 36 | Vanillylmandelic acid | 181.05 | 4.25 | 3.15 | 40.56 | 0.8 | 0.43 | ** |
| Organic acid | | | | | | | | |
| 37 | 2-Hydroxy-3-(4-hydroxyphenyl)propenoic acid | 222.08 | 4.59 | 2.17 | 12.89 | 1.17 | 0.78 | ** |
| 38 | 2-Propylglutaric acid | 216.12 | 3.48 | 3.3 | 36.09 | 1.49 | 1.95 | ** |
| 39 | 3,4-Dihydroxyphenylacetaldehyde | 363.11 | 4.06 | 4.69 | 21.5 | 0.15 | 0.66 | ** |
| 40 | 3-Hydroxyoctanoic acid | 479.32 | 3.57 | 6.03 | 45.06 | 1.27 | 2.32 | ** |
| 41 | 3-Isopropylmalic acid | 218.1 | 3.73 | 2 | 16.92 | 0.78 | 0.72 | ** |
| 42 | 4-Hydroxyphenylpyruvic acid | 179.03 | 3.75 | 5.9 | 45.18 | 3.47 | 2.33 | ** |
| 43 | Citric acid | 215.02 | 5.81 | 1.22 | 6.83 | 1.15 | 0.88 | ** |
| 44 | Glutaric acid | 174.08 | 5.06 | 0.7 | 9.32 | 0.92 | 0.84 | ** |
| 45 | Homovanillic acid | 224.09 | 3.48 | 3.59 | 36.17 | 1.37 | 1.95 | ** |
| 46 | Ketoleucine | 129.05 | 5.76 | 4.21 | 7.23 | 7.26 | 0.87 | ** |
| 47 | Pyruvic Acid | 175.02 | 3.91 | 0.72 | 18.96 | 3.95 | 0.69 | ** |
| 48 | Quinoline-4,8-diol | 162.05 | 5.09 | 3.16 | 8.17 | 0.89 | 1.16 | ** |
| Indole metabolites | | | | | | | | |
| 49 | 5-Hydroxyindoleacetate | 192.07 | 3.5 | 3.49 | 31.03 | 0.89 | 1.77 | ** |
| 50 | 5-Hydroxyindoleacetic acid | 209.09 | 6.26 | 2.43 | 7.91 | 1.06 | 1.16 | ** |
| 51 | 5-Hydroxyindoleacetylglycine | 249.09 | 5.05 | 4.17 | 29.23 | 1.46 | 1.71 | ** |
| 52 | Indole-3-acetic Acid | 158.06 | 4.35 | 2.05 | 17.41 | 0.47 | 1.38 | ** |
| 53 | Kynurenic Acid | 188.03 | 4.28 | 4.18 | 11.01 | 2.97 | 1.23 | ** |
| 54 | Xanthurenic Acid | 206.04 | 4.24 | 3.11 | 19.5 | 1.05 | 1.43 | ** |
| Vitamins and their derivatives | | | | | | | | |
| 55 | Biotin | 243.08 | 5.11 | 4.63 | 7.95 | 0.47 | 1.16 | ** |
| 56 | L-Ascorbic acid | 177.04 | 4.54 | 1.04 | 14.78 | 0.78 | 1.31 | ** |
| 57 | Pantothenic Acid | 218.1 | 6.41 | 3.43 | 7.33 | 1.54 | 1.15 | ** |
| 58 | Pyridoxal | 168.07 | 4.36 | 2.87 | 13.7 | 0.51 | 1.29 | ** |
| other | | | | | | | | |
| 59 | Atropaldehyde | 133.06 | 4.04 | 5.8 | 19.29 | 0.37 | 1.43 | ** |
| 60 | Morph | 292.13 | 5.33 | 3.55 | 14.08 | 8.56 | 1.3 | ** |

Note：P<0.05 is represented by *, P<0.01 is represented by **.

8. [Results]：9(S)-HODE and PA(8:0/a-15:0) are not commonly observed under metabolite and lipid profiling conditions. Have their peaks been confirmed, and have they been validated using standard compounds?

Thanks for your suggestions. Thanks to the editors for their attention to this technical detail, as you point out, 9(S)-HODE (hydroxylinotadienoic acid) and PA(8:0/a-15:0) (phosphatidic acid) are detected less frequently in routine metabolomic/lipidomic analyses. The peaks of both metabolites were detected and the picture is attached below. These two compounds have not been validated as commercial standards, mainly due to the scarcity of commercially available standards (e.g., PA(8:0/a-15:0) is not available) and the high cost of synthesis and customization. However, based on the following evidence, we believe that the identification results have high confidence. In this study, the non-targeted metabolomics strategy was used to identify metabolites by ion fragment search. The same substance has been found in other literature, cited in the following literature [1].

[1] LIU C M. Study on physiological changes and endocrine regulation of apple moth during diapause period [D]. Huazhong agricultural university, 2023. DOI: 10.27158 /, dc nki. Ghznu. 2023.000248.

Supporting Information Figure S5

Figure S5. Peak plot of 9(S)-HODE and PA(8:0/a-15:0) in *L.glycinivorella.*

9. In Figure S4, the colors and shapes of PCR and RNA-seq data are too similar, making them difficult to distinguish. It is necessary to represent them differently.

Thanks for your suggestions. We agree with you that PCR and RNA-seq data are really difficult to distinguish, and we will change the shape and color of PCR and RNA-seq data to facilitate better differentiation.

Supporting Information Figure S4

Figure S4. qRT-PCR validation of RNA-seq data of PD and D differential genes in *L.glycinivorella.*

10. The content of the following sentence does not match the figure legend, and the labels "A" and "B" are missing from Figure 3. Revisions are needed.

“50 of 107 were higher in PD (Figure 3B), and the top 5 up-regulated metabolites were Adenylosuccinate, 9(S)-HODE, PA(8:0/a15:0), N6-(1,2-Dicarboxyethyl)-AMP, and N-acetylaspartate. 57 of 107 were higher in D, and the top 5 down-regulated metabolites were 5-Hydroxy-L-tryptophan, PE (15:0/22:4(7Z,10Z,13Z,16Z)), Sphingosine, Pantothenic Acid, and Glycerophosphocholine.”

Thanks for your suggestions. We deeply regret the inconvenience caused to you by our negligence. In the resubmitted manuscript, we have corrected these legends that do not match the content, and added the legends of "A" and "B" in Figure 3. Thank you again for your correction.

Page 14, lines 309~314:

50 of 107 were higher in PD (Figure ~~3~~2B), and the top 5 up-regulated metabolites were Adenylosuccinate, 9(S)-HODE, PA(8:0/a-15:0), N6-(1,2-Dicarboxyethyl)-AMP, and N-acetylaspartate. 57 of 107 were higher in D (Figure 2B), and the top 5 down-regulated metabolites were 5-Hydroxy-L-tryptophan, PE (15:0/22:4(7Z,10Z,13Z,16Z)), Sphingosine, Pantothenic Acid, and Glycerophosphocholine.

Page 15, lines 327~334:

Using a thorough transcriptome and metabolomic study, the distinctions between the diapause and pre-diapause SPBs were further clarified. The O2PLS-DA was conducted, and the plots of the O2PLS-DA scores revealed a distinct divergence (Figure 3A). The combined KEGG pathway analysis showed that the changes were enriched in 352 pathways, with 5 pathways linked to the metabolism of amino acids, 3 pathways linked to the metabolism of carbohydrates, and 2 pathways linked to the metabolism of lipids in the top 20 (Table S12). Glycerophospholipid metabolism, sphingolipid metabolism, and tryptophan metabolism were the most abundant pathways (Figure 3B).

Figure 3. (A)O2PLS−Scores scatter diagram; (B) Integrated analysis of the top 20 KEGG pathways

11. It is difficult to find the level changes of DEGs in the paper through figures or tables. It is necessary to present the data more clearly for easier reference.

“These results showed that, in the developmental process from PD to D, most DEGs were related to the immune defense, energy metabolism and hormone synthesis. There were three downregulated DEGs related to insect immunity and defense (heat shock protein genes (HSP), Superoxide Dismutase (SOD), and catalase(CAT)), six down regulated DEGs related to energy metabolism (hexokinase (HK), pyruvate kinase (PK), ATP-dependent 6-phosphofructokinase (PFK), citrate synthase (CS), isocitrate dehydrogenase [NADP] cytoplasmic (IDH), α,α-trehalose-phosphate synthase [UDP forming] (TPS)), and two down-regulated DEGs related to hormone synthesis (juvenile hormone epoxide hydrolase (JHEH) and 20-hydroxyecdysone (20E)).”

“In this study, it was found that JHEH was down-regulated during diapause, thereby inhibiting the breakdown of juvenile hormones and keeping the larvae in the larval state for a long time, which may be the reason why SPB maintains diapause with mature larvae.”

Your suggestion is very insightful. Before we write the result of SOD, CAT, HSP, HK and PK, PFK, CS, IDH, TPS, introduces the JHEH etc, thank you for your careful check. After describing the pathways in which KEGG is significantly enriched, we have identified the significantly enriched genes in the top 10 pathways of enrichment, and arranged them in the form of a table attached below in order to clear the observed DEG associated with diapause. At the same time, the description of DEG is added in the text. We have provided detailed explanations of the changes made in the revised manuscript, including the specific locations where these changes can be found.

Page 11, lines 241~292:

KEGG pathways of DEGs could give deeper insights into the physiological processes related to diapause. Of all gene with a KEGG annotation, 2262 up-regulated DEGs were assigned to 318 KEGG pathways, and 5065 down-regulated DEGs were assigned to 336 KEGG pathways. Up-regulated DEGs were classified into “Ribosome biogenesis in eukaryotes”, “RNA degradation”, “Nucleotide excision repair”, and “RNA polymerase” (Figure 1C). Down-regulated DEGs were classified into “Glycolysis/Gluconeogenesis”, “Amino sugar and nucleotide sugar metabolism”, “Peroxisome”, and “Tryptophan metabolism” (Figure 1D). Top 10 KEGG pathways were significantly enriched, are listed in Table S7.

Enrichment with up-regulated differential genes Compared with the KEGG pathway, the number of pathways enriched by down-regulated differential genes was more significant. In the significantly upregulated genes, RPP25L (Ribonuclease P/MRP Subunit P25 Like), RIOK2 (Right Open Reading Frame Kinase 2), IMP3 (IGF2 mRNA-Binding Protein 3) Participation in Ribosome biogenesis in eukaryotes. RPP25L is a ribonuclease complex subunit that repairs damaged rRNA or tRNA and enhances stress resistance under low temperature or oxidative stress (32). RIOK2 is involved in cell cycle regulation and cell growth (33). IMP3 belongs to the insulin-like growth factor 2 mRNA binding protein family and usually plays a role in the growth, proliferation and differentiation of cells(34). It may be related to stress resistance in insect diapause.

~~These results showed that, in the developmental process from PD to D, most DEGs were related to the immune defense, energy metabolism and~~ ~~hormone synthesis. There were three down-regulated DEGs related to insect immunity and defense (heat shock protein genes (HSP), Superoxide Dismutase (SOD), and catalase(CAT)), six down-regulated DEGs related to energy metabolism (hexokinase (HK),~~ ~~pyruvate kinase (PK), ATP-dependent 6-phosphofructokinase (PFK), citrate synthase (CS), isocitrate dehydrogenase [NADP] cytoplasmic (IDH),~~ ~~α,α-trehalose-phosphate synthase [UDP-forming] (TPS)), and two down-regulated DEGs related to hormone synthesis (juvenile hormone epoxide hydrolase (JHEH) and 20-hydroxyecdysone (20E)).~~

Among the down-regulated differential genes, Hexokinase (HK) is involved in Glycolysis / Gluconeogenesis, amino and nucleotide glucose metabolism, and Galactose metabolism, catalyze the conversion of glucose to glucose 6-phosphate. Pyruvate Kinase(PK) is a key enzyme in glycolysis, and its activity changes affect energy metabolism. 6-phosphofructokinase (PFK) is a kinase One of the major rate-limiting enzymes, catalyzes the production of FDP (1, 6-diphosphate fructose) from Fractose 6-phosphate (F6P), controls the rate of glucose oxidation, and participates in Glycolysis / Gluconeogenesis. α,α-trehalose-phosphate synthase [UDP-forming] (TPS) is involved in starch and sucrose metabolism and is present in insects. An important enzyme in trehalose metabolism that breaks down trehalose into glucose. In addition, citrate synthase (CS), isocitrate dehydrogenase [NADP] cytoplasmic (IDH) were involved in the citric acid cycle, which were related to the energy metabolism of diapause. Catalase (CAT) and Superoxide Dismutase (SOD) participate in Peroxisome, Longevity regulating pathway-worm. heat shock protein genes (HSP) participate in Longevity regulating pathway-worm. They are closely related to oxidation in diapause larvae. Insect hormone biosynthesis juvenile hormone epoxide in the pathway hydrolase (JHEH) is an important enzyme in the insect hormone biosynthesis pathway, and the downregulation of this gene may cause the accumulation of juvenile hormone levels, which makes insects nutritious in larval state(Other differential genes are shown in Table S8).

Supporting Information Table S8.

TABLE S8. KEGG metabolic pathway and differentially expressed genes in *L.glycinivorella* during diapause.

| KEGG pathway | ID | Gene number | Differentially expressed genes | | P_value |
| --- | --- | --- | --- | --- | --- |
|  |  |  | up | down |  |
| Ribosome biogenesis in eukaryotes | map03008 | 44 | RPP25, IMP3, RIOK2, NOG1, UTP15, POP5, NHP2, NAN1, MPP10, REX1 |  | ** |
| RNA degradation | map03018 | 16 | RRP40, DCP2, LSM7, MTR3, ENO, WDR61 |  | ** |
| cleotide excision repair | map03420 | 14 | POLE3, XPC, TFIIH1, CETN2, ERCC4 |  | ** |
| RNA polymerase | map03020 | 14 | RPC8, RPA12, POLR2M, RPB1 |  | ** |
| Cytosolic DNA-sensing pathway | map04623 | 9 | TREX1, RPABC2, TMEM173, RPAC1 |  | ** |
| Glycolysis / Gluconeogenesis | map00010 | 34 |  | HK, FBP, GPI, PFK, PK, PGK, ALDH, ALDO, GAPDH, PGAM, MINPP1, ENO, PCK, DLAT, LDH, DLD, TPI | ** |
| Amino sugar and nucleotide sugar metabolism | map00520 | 34 |  | E3.2.1.14, HK, GMPP, CHS1, GPI, pgm, CYB5R, GMDS, HEXA_B, GALT, galK | ** |
| Peroxisome | map04146 | 36 |  | ACSL, CAT, E1.3.3.6, MPV17L, PEX7, XDH, HAO, FAR, ALDL1, PHYH, SOD, IDH1, XDH, FAR | ** |
| Tryptophan metabolism | map00380 | 23 |  | CAT, GCDH, TDO2, HADH, ALDH, KMO, HADHA, ECHS1, CCBL | ** |
| Longevity regulating pathway - worm | map04212 | 30 |  | CAT, SCD, VHL, GST, SMEK, FAR, IRS1, SOD2, DESAT1, GCLC, AKT, HSP, HIF1A | ** |
| Starch and sucrose metabolism | map00500 | 20 |  | HK, GPI, pgm, TPS, malZ, INV, AGL, G6PC, AGL, GYS, GAA, PYG, AMY | ** |
| Fatty acid degradation | map00071 | 19 |  | ACSL, GCDH, E1.3.3.6, ALDH, HADH, HADHA, ECHS1, ALDH7A1 | ** |
| Galactose metabolism | map00052 | 17 |  | HK, GALM, pgm, malZ, INV, G6PC, galK, PFK | ** |
| Citrate cycle (TCA cycle) | map00020 | 17 |  | LSC2, PDHB, MDH2, DLST, PC, SDHD, CS, IDH1, LSC1, ACLY, ACO, OGDH | ** |
| Insect hormone biosynthesis | map00981 | 17 |  | FOHSDR, ALDH, CYP18A1, JHEH, ALDH, SHD, FPPP | ** |

Note：P<0.05 is represented by *, P<0.01 is represented by **.

Noet：hexokinase type 2, HK; putative chitinase 1, E3.2.1.14; long-chain-fatty-acid--CoA ligase 5,ACSL; succinate--CoA ligase [ADP-forming] subunit beta, LSC2; catalase-like, CAT; pyruvate dehydrogenase E1 component subunit beta, PDHB; acyl-CoA Delta-9 desaturase-like, SCD; acidic mammalian chitinase-like, E3.2.1.14; glutaryl-CoA dehydrogenase, GCDH; peroxisomal acyl-coenzyme A oxidase 3, ACOX3; fructose-1,6-bisphosphatase 1, FBP; mpv17-like protein, MPV17L; tryptophan 2,3-dioxygenase, TDO2; pyruvate kinase-like, PK; aldehyde oxidase 4-like, XDH; chitin synthase chs-2, CHS1; malate dehydrogenase, mitochondrial, MDH2; glutathione S-transferase theta-1-like, GST; serine/threonine-protein phosphatase 4 regulatory subunit 3, SMEK; aldehyde dehydrogenase X, ALDH; glucose-6-phosphate isomerase, GPI; fatty acyl-CoA reductase wat-like, FAR; galactose mutarotase-like, GALM; cytochrome P450 18a1, CYP18A1; probable 3-hydroxyacyl-CoA dehydrogenase B0272.3, HADH; phosphoglucomutase, pgm; ATP-binding cassette sub-family D member-like, ABCD2; NADH-cytochrome b5 reductase 2, transcript variant X1, CYB5R; juvenile hormone epoxide hydrolase-like, JHEH; aldehyde dehydrogenase 1A1-like, ALDH; pyruvate carboxylase, PC; alpha,alpha-trehalose-phosphate synthase [UDP-forming]-like, TPS; maltase A1-like, malZ; peroxisomal multifunctional enzyme type 2-like, HSD17B4; GDP-mannose 4,6 dehydratase, transcript variant X1, GMDS; probable alpha-ketoglutarate-dependent hypophosphite dioxygenase, PHYH; superoxide dismutase [Mn], SOD2; fructose-bisphosphate aldolase, ALDO; probable citrate synthase 2, mitochondrial, CS; peroxiredoxin-5, mitochondrial, PRDX5; acyl-CoA Delta(11) desaturase-like, DESAT1; phosphatidylinositol 4,5-bisphosphate 3-kinase catalytic subunit delta isoform, PIK3CA_B_D; triosephosphate isomerase, TPI; beta-hexosaminidase subunit alpha-like, HEXA_B; glutathione S-transferase 1-like, transcript variant X1, GST; tropinone reductase-like 3, DHRS4; N-acetylneuraminate lyase-like, NPL; RAC serine/threonine-protein kinase, AKT; phosphoglycerate mutase 1, PGAM; delta(3,5)-Delta(2,4)-dienoyl-CoA isomerase, mitochondrial, ECH1; glucose-6-phosphatase 3, G6PC; 1,4-alpha-glucan-branching enzyme, GBE1, glgB; ATP-dependent 6-phosphofructokinase, PFK; phosphoglycolate phosphatase 1A, chloroplastic-like, FPPP; galactokinase-like, galK; acidic mammalian chitinase-like, E3.2.1.14; DNA polymerase epsilon subunit 3, POLE3; U3 small nucleolar ribonucleoprotein protein IMP4, IMP4; HEAT repeat-containing protein 1 homolog, UTP10; exonuclease DPD1, TREX1; ribonuclease P protein subunit p25-like protein, RPP25; replication protein A 32 kDa subunit, RFA2;adenylate kinase isoenzyme 6 homolog, AK6; DNA repair protein complementing XP-C cells homolog, XPC;DNA-directed RNA polymerase I subunit RPA12, RPA12, POLR1H, ZNRD1; DNA-directed RNA polymerase II subunit Rpb4, RPB4, POLR2D; nucleolar GTP-binding protein 1, transcript variant X1, NOG1; enolase-like, transcript variant X2, ENO; U6 snRNA-associated Sm-like protein LSm6, LSM6; DNA repair endonuclease XPF, ERCC4, XPF; stimulator of interferon genes protein-like, TMEM173, MITA; probable RNA 3'-terminal phosphate cyclase-like protein, RCL1; general transcription factor IIH subunit 1, TFIIH1, GTF2H1, TFB1; DNA polymerase delta subunit 3, POLD3; U3 small nucleolar RNA-associated protein 15 homolog, UTP15; WD repeat-containing protein 36, UTP21, WDR36; serine/threonine-protein kinase RIO2, RIOK2; DNA-directed RNA polymerases I and III subunit RPAC1, RPAC1, RPC40, POLR1C; ribonuclease P/MRP protein subunit POP5, transcript variant X1, POP5; DNA-directed RNA polymerase I subunit RPA49-like, RPA49, POLR1E; ribosomal RNA small subunit methyltransferase NEP1, EMG1, NEP1; M-phase phosphoprotein 6, MPHOSPH6, MPP6; ribosome biogenesis protein BMS1 homolog, BMS1; U3 small nucleolar ribonucleoprotein protein IMP3, IMP3.

12. [Discussion]：Please provide the evidence based on the results to support the conclusion stated in the sentence on page 17, line 253 of the Discussion section.

“it is of great significance to study the transcriptome and metabolome of soybean insect at different diapause stages.”

Thanks for your comments. We will expand the discussion of this section to include a more detailed analysis of the experimental results, which have been supplemented to the original manuscript, as shown below. Further comparison with recent research in the field will strengthen the overall argument and ensure that the manuscript meets the criteria of a journal (2).

(2) Wang J, Fan H, Xiong KC, Liu YH. Transcriptomic and metabolomic profiles of Chinese citrus fly, Bactrocera minax (Diptera: Tephritidae), along with pupal development provide insight into diapause program. Hull JJ, editor. PLoS ONE. 2017 Jul 12;12(7):e0181033.

Page 16, lines 354~370:

Diapause strategy is a key determinant for insect survival under seasonal extreme environmental conditions. Considering that soybean insect can regulate its physiological state by timely changing gene expression and metabolite levels to achieve homeostasis, under low temperature stress. Through integrated transcriptomic and metabolomic analyses of SPB, genes associated with diapause were identified. There were three down-regulated DEGs related to insect Antioxidase regulation (heat shock protein genes (HSP), Superoxide Dismutase (SOD), and catalase(CAT)), six down-regulated DEGs related to energy metabolism (hexokinase (HK), pyruvate kinase (PK), ATP-dependent 6-phosphofructokinase (PFK), citrate synthase (CS), isocitrate dehydrogenase [NADP] cytoplasmic (IDH), α, α-Trehalose-phosphate synthase [UDP-forming] (TPS)), and two down-regulated DEGs related to hormone synthesis (juvenile hormone epoxide hydrolase (JHEH) and 20-hydroxyecdysone (20E)). Finding the metabolites associated with diapause: glycerophospholipid, pyruvate, and arginine, proline, laid the foundation for the theoretical study of the molecular mechanism of diapause, for further interferenceThe diapause method can provide reference for its prevention and treatment. it is of great significance to study the transcriptome and metabolome of soybean insect at different diapause stages.

13. If there are existing studies related to the following sentence, relevant references should be added.

“Certain metabolites, such as Phosphatidyl choline (PC) and proline, can act as cryoprotectants to improve cold tolerance in diapausing insects.”

We sincerely appreciate the valuable comments.We have checked the literature carefully and added more references on Phosphatidyl choline (PC) and proline into the Discussion part in the revised manuscript.

Page 16, lines 354~355:

Certain metabolites, such as Phosphatidyl choline (PC) and proline, can act as cryoprotectants to improve cold tolerance in diapausing insects(44-46).

44.	Teets NM, Denlinger DL. Physiological mechanisms of seasonal and rapid cold‐hardening in insects. Physiol Entomol. 2013 Jun;38(2):105–16.

45.	Huang Q, Ma Q, Li F, Zhu-Salzman K, Cheng W. Metabolomics Reveals Changes in Metabolite Profiles among Pre-Diapause, Diapause and Post-Diapause Larvae of Sitodiplosis mosellana (Diptera: Cecidomyiidae). Insects. 2022 Mar 30;13(4):339.

46.	Batz ZA, Armbruster PA. Diapause-associated changes in the lipid and metabolite profiles of the Asian tiger mosquito, Aedes albopictus. J Exp Biol. 2018 Dec 13;221(Pt 24):jeb189480.

14. Figure 4 appears to represent the key metabolic pathways identified in this study. Therefore, it is necessary to provide an interpretation and discussion of the key metabolites and transcriptomic changes observed in the study based on the measured results. Including unverified metabolites and transcripts in the interpretation may lead to overstatements. For example, regarding glycerol aldehyde 3-phosphate (G3P), the discussion includes changes in glycerol kinase (GK), glucose, glycogen, and glycerol. It is important to specify whether changes in glycerol kinase (GK), glucose, glycogen, and glycerol were actually observed.

Thanks for your suggestions. Figure 4 represents the key metabolic pathways identified in this study. Therefore, we interpret the key metabolite and transcriptomic changes observed in the study based on your suggestions and measurements, including the source pathways of differential genes and metabolites. In this study, no significant differences in metabolites such as glycogen, glucose and glycerol were detected, but significant changes in transcription factors(glycerol kinase (GK), glycogen synthase (GYS)) were found in this process, conjecturing that the TCA cycle and glycolysis process are dynamic changes, The entire metabolic network is in homeostasis.

Page 16, lines 374~423:

Certain transcription factors such as Trehalose, due to its unique chemical properties, is the main sugar circulating in the hemolymph of most insects and is important for protecting cells from various environmental stresses (43-45) trehalose-phosphate synthase (TPS), and trehalose 6-phosphate phosphatase (TPP) were significantly down-regulated and caused trehalose accumulation, which is reflected in the low energy metabolism maintained in SPB. ~~Glycogen is thought to be converted to polyhydroxyl alcohols or trehalose in diapause insects. Glycogen phosphorylase (GP) catalyzes the phosphorylation that starts this reaction. In one instance, phosphoglucose interchemase (PGM) transforms it into D-glucose-6-phosphate. Glycolysis can transform D-glucose-6-phosphate into pyruvate, which then enters the TCA cycle. Hexokinase (HK) and pyruvate kinase (PK), citrate synthase (CS) of the TCA cycle pathway, isocitrate dehydrogenase (IDH),~~ Glycolysis is the most primitive way for cells to obtain energy, and it is also the main pathway in glucose catabolism. As a key enzyme in the first rate-limiting reaction of glycolysis pathway, hexokinase (HK) is widely present in organisms and can catalyze the conversion of glucose to glucose 6-phosphate. 6-phosphofructokinase (PFK) can catalyze the production of FDP from Fractose 6-phosphate (F6P). Its activity can be regulated according to the energy requirements of the cell, controlling the rate of glucose oxidation. In this study, the contents of HK, PK and PFK were significantly reduced, which is presumed to be related to the accumulation of metabolite glucose. The TCA cycle is a common metabolic way to obtain energy in aerobic organisms. In this study, citrate synthase (CS), isocitrate dehydrogenase (IDH) and malate dehydrogenase (MDH) were significantly down-regulated, indicating that the cycle rate of energy supply in insects was significantly lower Metabolic levels are reduced in response to adverse conditions. In summary, both the TCA cycle and glycolysis synergistically reduce activity to reduce energy consumption.

Glucose can also be converted into glycerol, which can be used as an anti-freezing protective agent to enhance the cold tolerance of insects (27). Glycerol kinase (GK) is a rate-limiting enzyme in glycerolipid metabolism by the conversion of glycerol aldehyde 3-phosphate (G3P) to phospholipids ~~such as glyceryl phosphate~~. The catabolism of glycerol in vivo consists of a two-step reaction, in which glycerol is catalyzed by glycerol kinase and phosphorylated to produce glycerol 3-phosphate. Rapid cold acclimation of *Sarcophaga crassipalpis* and *Spodoptera exigua* showed that they acted as the main anti-freeze protective substances by increasing their glycerol content(28). During diapause, GK was significantly down-regulated, This indicates the accumulation of glycerol in SPB, suggesting that this enzyme is mainly used as a protective substance to enhance the cold resistance of insects to survive diapause environment. As a key core factor in insulin signaling pathway, PDK1 phosphorylates and activates serine/threonine protein kinase (AKT), thereby regulating glucose homeostasis (48). An important gene downstream of AKT is glycogen synthase kinase 3β (GSK3β), which is involved in glycogen biosynthesis and metabolism. GSK3β inhibits glycogen synthase by phosphorylating glycogen synthase (GYS). In this way, it inhibits glycogen biosynthesis (49). In this study, by inhibiting GSK3β, AKT was able to overcome this inhibition and increase GYS activity, thereby affecting glycogen content. In this study, no significant differences in metabolites such as glycogen, glucose and glycerol were detected, but significant changes in transcription factors were found in this process, conjecturing that the TCA cycle and glycolysis process are dynamic changes, The entire metabolic network is in homeostasis.

15. The title "4.2 Immune Response in the Diapause" primarily refers to immune response; however, the content mainly discusses ROS and antioxidants. A more appropriate revision of the title is needed to accurately reflect the content.

Thank you for pointing this out. We believe that Antioxidase regulation is a better place to discuss reactive oxygen species (ROS) and antioxidants. Now change the title to Antioxidase regulation.

Page 19, lines 445:

4.2 ~~immune response~~ Antioxidant regulation in the diapause

16. [Minor]：Spelling corrections are needed throughout various sections of the paper.

We have carefully examined the full text and have corrected it accordingly. These errors did affect the overall quality of the article, for which we apologize and sincerely thank you for pointing them out. In the revised draft, we have fixed terminology errors (e.g. "univoltine", "PTTH"), unified citation format (add Spaces), and unified terminology (e.g. "soybean pod borer" and abbreviation SPB); Optimized sentence logic (e.g. splitting long sentences, adjusting redundant expressions); Enhance academic rigor (e.g. avoid absolutist statements such as "will be revealed").

Page 1, lines 16~39:

*Leguminivora glycinivorella* is a major pest in soybean production, causing huge economic losses to soybean production when the damage is severe, It typically usually overwintering with mature larvae lagging, which may be an effective tool for pest management. In this study, the key substances of diapause regulation were identified by differential expression genes and differential metabolites, using transcriptome and metabolomics analyses in the diapause and pre-diapause stages in *L. glycinivorella*. The findings revealed that 5558 genes in ~~all~~ total were significantly ~~changed~~ altered during diapause, ~~and~~ with pyruvate kinase (PK), trehalose synthase (TPS), superoxide dismutase (SOD), citrate synthase (CS), and 20-hydroxyecdysone (20E) ~~were significantly decreased during diapause~~ showing significant decreases. There were 1628 metabolites with significant changes, especially proline, Phosphatidyl choline, and unsaturated fatty acids increased significantly in the period of diapause. Meanwhile, KEGG analysis ~~basing~~ based on the above differential substances showed that they were involved in TCA cycle, glycolysis, ~~insulin signaling pathway,~~ and glycerophospholipid metabolic pathway, which suggesting that they may be closely related to energy reserve, Antioxidase regulation ~~immune regulation~~ and hormone regulation during diapause. In this study, we present a comprehensive ~~transcriptome and metabolome~~ transcriptomic and metabolomic analysis that identifies three important molecular events during diapause (energy reserve, immune enhancement, hormone regulation) that may play a role in survival and stress resistance during diapause. These findings have greatly improved our understanding of diapause of *L. glycinivorella*, provided a theoretical basis for clarifying the molecular mechanism of diapause regulation of *L. glycinivorella*, and further improved the level of prediction and comprehensive management of *L. glycinivorella*.

Reviewer #2: General comments:

This study investigates the molecular basis of diapause in Leguminivora glycinivorella, a major soybean pest responsible for significant economic losses. By integrating transcriptomic and metabolomic analyses, the authors compared gene expression and metabolite profiles between pre-diapause and diapause stages, identifying differentially expressed genes and significantly altered metabolites. The findings highlight three critical molecular events during diapause—energy reserve accumulation, immune enhancement, and hormonal regulation—which collectively serve as adaptive mechanisms for survival under stress conditions. These findings advance understanding of diapause regulation in L. glycinivorella.

Major comments:

1. All figures in the manuscript are of insufficient resolution, making it difficult to discern the details and interpret the data accurately.

Thank you for your advice. The resolution of all the images in the manuscript has been revised and submitted in the mail, and will be submitted together with the revised manuscript in order to identify details and accurately interpret the data.

2. The qPCR results are not adequately described in the main text, and their significance is unclear.

Thank you for pointing this out. We have supplemented the results of QPCR and its significance according to the Reviewer's suggestion.

Page 14, lines 293~314:

To verify the reliability of the transcriptome sequencing results, six differentially expressed genes were selected for real-time fluorescence quantitative PCR verification, three of which were significantly up-regulated genes from Ribosome biogenesis in eukaryotes. Three significantly down-regulated genes were selected from Peroxisome and Citrate cycle (TCA cycle). Results The expression trend of these 6 differentially expressed genes between pre-diapause group and diapause group was found in transcriptome and real-time fluorescence quantitative PCR. The consistency indicates that the transcriptome sequencing results are reliable (Figure S4) .

3.2 Metabolome analyses

Using UHPLC-MS/MS, 1628 metabolites were found in all SPB samples at two physiological stages linked to PD and D. It was determined that these detected metabolites corresponded to nine different biochemical categories (Table S9). 1628 metabolites were subjected to principal component analysis (PCA), which clearly distinguished between D and PD (Fig. 2A). Pairwise comparisons revealed that 107 of these metabolites were differential metabolites. along with a two-way heat map with a hierarchical clustering diagram (Figure 2B) and related data displayed the top ten of these differential metabolites (Table S10). 50 of 107 were higher in PD (Figure ~~3~~2B), and the top 5 up-regulated metabolites were Adenylosuccinate, 9(S)-HODE, PA(8:0/a-15:0), N6-(1,2-Dicarboxyethyl)-AMP, and N-acetylaspartate. 57 of 107 were higher in D (Figure 2B), and the top 5 down-regulated metabolites were 5-Hydroxy-L-tryptophan, PE (15:0/22:4(7Z,10Z,13Z,16Z)), Sphingosine, Pantothenic Acid, and Glycerophosphocholine.

3.Additionally, the genes IMP3, RIOK2, and RPP25L mentioned in Figure S4 and Table S1 are not introduced or discussed.

Thanks for your careful checks. We are sorry for our carelessness. the genes IMP3, RIOK2, and RPP25L mentioned in Figure S4 and Table S1 are presented and discussed below.

Page 11, lines 241~263:

KEGG pathways of DEGs could give deeper insights into the physiological processes related to diapause. Of all gene with a KEGG annotation, 2262 up-regulated DEGs were assigned to 318 KEGG pathways, and 5065 down-regulated DEGs were assigned to 336 KEGG pathways. Up-regulated DEGs were classified into “Ribosome biogenesis in eukaryotes”, “RNA degradation”, “Nucleotide excision repair”, and “RNA polymerase” (Figure 1C). Down-regulated DEGs were classified into “Glycolysis/Gluconeogenesis”, “Amino sugar and nucleotide sugar metabolism”, “Peroxisome”, and “Tryptophan metabolism” (Figure 1D). Top 10 KEGG pathways were significantly enriched, are listed in Table S7.

Enrichment with up-regulated differential genes Compared with the KEGG pathway, the number of pathways enriched by down-regulated differential genes was more significant. In the significantly upregulated genes, RPP25L (Ribonuclease P/MRP Subunit P25 Like), RIOK2 (Right Open Reading Frame Kinase 2), IMP3 (IGF2 mRNA-Binding Protein 3) Participation in Ribosome biogenesis in eukaryotes. RPP25L is a ribonuclease complex subunit that repairs damaged rRNA or tRNA and enhances stress resistance under low temperature or oxidative stress(32).RIOK2 is involved in cell cycle regulation and cell growth(33). IMP3 belongs to the insulin-like growth factor 2 mRNA binding protein family and usually plays a role in the growth, proliferation and differentiation of cells(34). It may be related to stress resistance in insect diapause.

4.Furthermore, there is a repetition in the listing of the RPP25L-F primer sequences, which needs to be corrected.

We feel sorry for our carelessness. In our resubmitted manuscript, RPP25L primer sequences have been completed.Thanks for your correction.

Supporting Information TABLE S2.

| Unigene ID | Forward primer |
| --- | --- |
| actin-F | 5' TCTTCCAGCCCTCGTTCC 3' |
| actin-R | 5' TTGTTGGCATACAGGTCCTTAC 3' |
| SOD-F | 5' CCAGACCTGCCGTATGAG 3' |
| SOD-R | 5' TGTCAATGTCGCCTTTAGC 3' |
| CS-F | 5' AGTTCGCCCTGAAGCACC 3' |
| CS-R | 5' CAGCCCGTAATACTGTAGAAGG 3' |
| IDH-F | 5' GGAAAGGTGGAGTTGGTGTA 3' |
| IDH-R | 5' CCGTCATATCGCTTGAGAAT 3' |
| IMP3-F | 5' CCGTGAGGTAGCGAACAA 3' |
| IMP3-R | 5' TGACCGGCTGACCAGGAA 3' |
| RIOK2-F | 5' CGTCCATTGCGAATCTCC 3' |
| RIOK2-R | 5' CCACGCCTATCTGGTTGC 3' |
| RPP26L-F | 5' TTGTAAGCGAGTTTGGTA 3' |
| RPP25L-R | 5' AAAGGTGGAAGTAAGATG 3' |

5. While KEGG pathway enrichment analysis identifies key pathways such as the TCA cycle and glycolysis, the study fails to explain how these pathways interact to regulate diapause. For instance, it remains unclear whether energy conservation during diapause is achieved through suppressed glycolysis or enhanced lipid metabolism.

We thank the reviewers for this suggestion, which really adds a new perspective to our research. Firstly, diapause is a period of programmed developmental arrest regulated by environmental factors, and its main feature is the obvious inhibition of metabolism. The results of this study showed that the contents of HK, PK and PFK in the glycolysis pathway were significantly reduced, indicating that the glycolysis process was weakened and the life activity was maintained at a low level, so as to improve its stress resistance. In The TCA cycle, citrate synthetase (CS), isocitrate dehydrogenase (IDH) and malate dehydrogenase (MDH) were significantly down-regulated, indicating that the energy supply cycle rate of insects was significantly reduced. The metabolite pyruvate, which is an intermediate linking glycolysis and TCA cycles, is significantly down-regulated during diapause，suggesting that the TCA cycle and glycolysis synergistically reduce activity to reduce energy consumption. In lipid metabolism, the contents of unsaturated fatty acids such as 13(S)-HODE and Lysophospholipids were significantly upregated, which made SPB resist the stress of low temperature. Lipid metabolism compensates for the deficiency of glucose metabolism through slow but persistent energy supply patterns. This dynamic balance of metabolic networks allows organisms to survive extreme resource constraints while retaining the flexibility to recover. The revisions to the revised draft are as follows.

Page 17, lines 371~401:

The accumulation of energy reserves is a prerequisite for the survival of insects during the diapause period. Almost all insects accumulate nutrients in their bodies (39–43). Certain metabolites, such as Phosphatidylcholine (PC) and proline, can act as cryoprotectants to improve cold tolerance in diapausing insects (44–46). Certain transcription factors such as Trehalose, due to its unique chemical properties, is the main sugar circulating in the hemolymph of most insects and plays a critical role in protecting cells from environmental stress (47–49). trehalose-phosphate synthase (TPS), and trehalose 6-phosphate phosphatase (TPP) were significantly down-regulated and caused trehalose accumulation, which is reflected in the low energy metabolism maintained in SPB. ~~Glycogen is thought to be converted to polyhydroxyl alcohols or trehalose in diapause insects. Glycogen phosphorylase (GP) catalyzes the phosphorylation that starts this reaction. In one instance, phosphoglucose interchemase (PGM) transforms it into D-glucose-6-phosphate. Glycolysis can transform D-glucose-6-phosphate into pyruvate, which then enters the TCA cycle. Hexokinase (HK) and pyruvate kinase (PK), citrate synthase (CS) of the TCA cycle pathway, isocitrate dehydrogenase (IDH),~~Glycolysis is the most primitive way for cells to obtain energy, and it is also the main pathway in glucose catabolism. As a key enzyme in the first rate-limiting reaction of glycolysis pathway, hexokinase (HK) is widely present in organisms and can catalyze the conversion of glucose to glucose 6-phosphate. 6-phosphofructokinase (PFK) can catalyze the production of FDP from Fractose 6-phosphate (F6P). Its activity can be regulated according to the energy requirements of the cell, controlling the rate of glucose oxidation. In this study, the contents of HK, PK and PFK were significantly reduced, which is presumed to be related to the accumulation of metabolite glucose. The TCA cycle is a common metabolic way to obtain energy in aerobic organisms. In this study, citrate synthase (CS), isocitrate dehydrogenase (IDH) and malate dehydrogenase (MDH) were significantly down-regulated, indicating that the cycle rate of energy supply in insects was significantly lower Metabolic levels are reduced in response to adverse conditionsThe metabolite pyruvate, which is an intermediate linking glycolysis and TCA cycles, is significantly down-regulated during diapause，suggesting that the TCA cycle and glycolysis synergistically reduce activity to reduce energy consumption.

6. Some of the bioinformatic tools, such as HISAT, StringTie, and DESeq2, lack reference citations.

According to the reviewer's suggestion, we carefully checked the literature and added Some references for Some of the bioinformatic tools, such as HISAT, StringTie, and DESeq2 in the revised draft.（29-31）

Page 7, lines 144~150:

Clean reads in orientation mode were individually aligned to the reference genome using HISAT2 software (29)

Using a reference-based process, StringTie put together each sample's mapped reads (30).

The analysis of differential expression was done with DESeq2 (31)

Genes with FDR="0.05" and |log2FC|≧1 were identified as substantially divergent expressed genes (DEGs) (32).

29.	Kim D, Langmead B, Salzberg SL. HISAT: a fast spliced aligner with low memory requirements. Nat Methods. 2015 Apr;12(4):357–60.

30.	Pertea M, Pertea GM, Antonescu CM, Chang TC, Mendell JT, Salzberg SL. StringTie enables improved reconstruction of a transcriptome from RNA-seq reads. Nat Biotechnol. 2015 Mar;33(3):290–5.

31.	Wang L, Feng Z, Wang X, Wang X, Zhang X. DEGseq: an R package for identifying differentially expressed genes from RNA-seq data. Bioinformatics. 2010 Jan 1;26(1):136–8.

32.	The genetic architecture of odor-guided behavior in Drosophila: epistasis and the transcriptome. Nature genetics. 2003;35(2):180–4.
